# Enhanced Methodology for Peptide Tertiary Structure Prediction Using GRSA and Bio-Inspired Algorithm

**DOI:** 10.3390/ijms26157484

**Published:** 2025-08-02

**Authors:** Diego A. Soto-Monterrubio, Hernán Peraza-Vázquez, Adrián F. Peña-Delgado, José G. González-Hernández

**Affiliations:** 1 Instituto Politécnico Nacional, Centro de Investigación en Ciencia Aplicada y Tecnología Avanzada, Km.14.5 Carretera Tampico-Puerto Industrial Altamira, Altamira 89600, Tamaulipas, Mexico; 2 Departamento de Mecatrónica y Energías Renovables, Universidad Tecnológica de Altamira, Boulevard de los Ríos Km. 3 + 100, Puerto Industrial Altamira, Altamira 89601, Tamaulipas, Mexico; apea@utaltamira.edu.mx (A.F.P.-D.); jggonzalez@utaltamira.edu.mx (J.G.G.-H.)

**Keywords:** peptide structure prediction, golden ratio simulated annealing, bio-inspired algorithm, metaheuristics

## Abstract

Recent advancements have been made in the precise prediction of protein structures within the Protein Folding Problem (PFP), particularly in relation to minimizing the energy function to achieve stable and biologically relevant protein structures. This problem is classified as NP-hard within computational theory, necessitating the development of various techniques and algorithms. Bio-inspired algorithms have proven effective in addressing NP-hard challenges in practical applications. This study introduces a novel hybrid algorithm, termed GRSABio, which integrates the strategies of Jumping Spider Algorithm (JSOA) with the Golden Ratio Simulated Annealing (GRSA) for peptide prediction. Furthermore, the GRSABio algorithm incorporates a Convolutional Neural Network for fragment prediction (FCNN), forms an enhanced methodology called GRSABio-FCNN. This integrated framework achieves improved structure refinement based on energy for protein prediction. The proposed enhanced GRSABio-FCNN approach was applied to a dataset of 60 peptides. The Wilcoxon and Friedman statistics test were employed to compare the GRSABio-FCNN results against recent state-of-the-art-approaches. The results of these tests indicate that the GRSABio-FCNN approach is competitive with state-of-the-art methods for peptides up to 50 amino acids in length and surpasses leading PFP algorithms for peptides with up to 30 amino acids.

## 1. Introduction

Proteins are composed of a linear sequence of amino acids and are essential for the biological functions of living organisms. To develop their biological function, proteins must adopt their native structure (NS), a unique three-dimensional conformation. A protein consists of one or more polypeptides, which are chains formed by multiple amino acids. The linkage of two or more amino acids through peptide bonds results in the formation of a peptide. Longer chains of amino acids are generally referred to as proteins, whereas shorter chains are classified as peptides. Peptides play significant roles in various physiological functions [1] and have demonstrated potential health benefits [2], along with applications in medicine [3] and biomedical research [4,5].

The Protein Folding Problem (PFP) seeks to predict a protein’s three-dimensional NS solely from its amino acid sequence. Accurate prediction of protein structures is crucial for understanding their biological function, drug design, and advancements in biotechnological applications [6,7,8]. Computational methods frequently rely on assembling fragments from known protein structures to predict the conformation of a target protein [9,10]. However, the vast conformational space and the intricate interplay of physical, chemical, and biological forces render this a complex challenge. Moreover, due to its complexity, the PFP is considered an NP-hard problem [11], as no known exact algorithms can solve it in polynomial time, and finding a solution is computationally intensive.

One effective approach to solving NP-hard problems is through metaheuristic algorithms. These algorithms leverage diversification to explore multiple local solutions while avoiding entrapment in local optima. Consequently, metaheuristics have been successfully applied to address complex challenges across various fields, including engineering, industry, energy, and bioinformatics [12,13,14,15].

Metaheuristics can be classified into the following categories [16]: evolutionary, physics-based, chemistry-based, human-based, mathematical-based, swarm-based, and bio-inspired algorithms.

Bio-inspired algorithms draw inspiration from natural processes and biological systems to develop optimization techniques. These algorithms mimic behaviors observed in nature, such as evolution, swarm intelligence, and immune responses, to solve complex computational problems. Relevant examples in the literature that utilize these behaviors include, among others, the Genetic Algorithm (GA) mimics natural selection by employing mutation, crossover, and selection to evolve solutions over generations [17]; and Particle Swarm Optimization (PSO), inspired by the collective behavior of birds and fish, adjusts individual agents’ positions based on personal and group experiences to find optimal solutions [18]. Similarly, Ant Colony Optimization (ACO) models ant behavior, using pheromone trails to identify optimal paths, making it effective for routing and scheduling problems [19,20]. Other examples of bio-inspired algorithms are the Dingo Optimization Algorithm (DOA) [21], Black widow Optimization Algorithm (BWOA) [22], Coot Bird Algorithm (COOT) [23], Mexican Axolotl Optimization (MAO) [24], Horned Lizard Optimization Algorithm (HLOA) [25], and Ant Lion Optimizer (ALO) [26].

Physics- and chemistry-based algorithms utilize fundamental physical laws and chemical interactions to model, simulate, optimize, and predict the behavior of complex systems. They are widely used in fields such as molecular biology, materials science, drug design, and energy research. For instance, Simulated Annealing (SA) mimics the gradual cooling process in metallurgy, accepting worse solutions early to escape local optima [27]; Gravitational Search Algorithm (GSA) uses Newton’s law of gravity to guide the population towards optimal solutions [28]. Chemical Reaction Optimization (CRO) simulates molecular interactions and transformations using decomposition, synthesis, and collision operators to explore the solution space [29]. These algorithms are particularly effective for addressing global optimization problems. Other notable examples include the Big Bang-Big Crunch (BB-BC) Algorithm [30], Thermal Exchange Optimization (TEO) [31], and Artificial Chemical Reaction Algorithm (ACRA) [32].

Several bio-inspired, physics-based, and chemical-based algorithms have also been applied to the PFP, including Rank-Based Ant Colony Optimization (RBCOA) [33], Gravitropism Artificial Plant Optimization Algorithm (GAPOA) [34], Cuckoo Search Algorithm (CSA) [35], Simulated Annealing (SA) [36], and Molecular Structure-Based Optimization (MSBO) [37]. The combination of bio-inspired, physics-based, chemistry-based, or other types of metaheuristics results in hybrid approaches that enhance overall performance [38]. For this reason, the hybridization of metaheuristic algorithms can enhance existing methods by producing higher-quality solutions. In general, metaheuristics algorithms have been successfully incorporated into methods applied to proteins or peptides, such as AlphaFold2 [39], Rosetta [40], GRSA2-SSP [41], GRSA2-FCNN [42], and PEP-FOLD3 [43].

In this work, we propose a new metaheuristic, GRSABio, a novel hybridization algorithm, in which Golden Ratio Simulated Annealing (GRSA) integrates strategies of the Jumping Spider Optimization Algorithm (JSOA), a bio-inspired algorithm, to enhance optimization performance. JSOA is inspired by the hunting habits of Arachnida Salticidae (jumping spiders), mimicking the spiders’ natural behaviors and mathematically modeling their hunting strategies, including searching, pursuing, and jumping to capture prey [44]. These behaviors provide a well-balanced approach between exploitation and exploration of the solution search space. We integrated GRSABio into a fragment-based methodology for protein structure prediction. Fragment-based methods build a protein structure by assembling short fragments derived from known structures, a widely used method in the literature [45]. In this work, the fragments are generated through a fragment prediction process using a Convolutional Neural Network (FCNN). The integration of the GRSABio algorithm with the FCNN results in an enhanced methodology named GRASBio-FCNN. This methodology was evaluated using structural metrics, including RMSD, GDT-TS, and TM-score [46]. Additionally, we apply the Wilcoxon signed-rank and Friedman tests to compare our methodology against the most popular algorithms in the literature.

## 2. Results

We conducted an experiment and evaluation with the proposed GRSABio algorithm, integrated into the GRSABio-FCNN methodology, using a set of 60 peptides (instances) sourced from the literature [41,42,47,48,49]. We compared its performance to GRSA2-FCNN [42], PEP-FOLD3 [43], AlphaFold2 [39], I-TASSER [50], Rosetta [40], and TopModel [51]. The instances used in the experiment include peptides with amino acid (residue) lengths ranging from 9 to 49, based on their primary structure. Consequently, the variation in torsion angles spans from 47 to 304 for each peptide. Table 1 presents the characteristics of each instance, including the PDB code from the Protein Data Bank (PDB) [52], the number of amino acids (aa), the number of torsion angles (Variables), the type of Secondary Structure (SS), and the experimental solution method (Method) used to obtain the native structure (NS) and recorded in the PDB. The experimentation consists of two evaluations. The first evaluation involves four groups of 15 instances each. The second evaluation classifies the dataset based on secondary structure types to analyze the behavior of each algorithm concerning its corresponding secondary structure type. For this, the dataset is categorized into three groups: alpha (mostly alpha-helical), beta (mostly beta-sheet), and none (lacking a dominant alpha or beta structure).

The GRSABio algorithm was executed 30 times for each instance. The software used for this experimentation included the SMMP package (version 3.0) in FORTRAN for protein structure calculations using the ECEPP/2 energy function, and Python (version 3.13) for extracting peptide structure fragments. GRSABio was run on a generic computing system at IPN-CICATA Altamira, which has the characteristics: 13th Gen Intel ^®^ Core i9-13900K processor running at 5.8 GHz, memory: 192 GB of RAM, and the Linux Ubuntu 24.04 LTS operating system. Table 2 presents the parameter settings for all algorithms.

Our methodology was evaluated using structural metrics, including the TM-score [53], Global Distance Test-Total Score (GDT-TS) [54], and Root Mean Square Deviation (RMSD) [55]. The TM-score and GDT-TS range from 0 to 1, measuring the similarity between two protein structures. For the TM-score, a value exceeding 0.5 and approaching 1 indicates high structural similarity, whereas for RMSD, lower values closer to 0 signify better alignment. In the CASP competition, these metrics are commonly employed to evaluate the quality of PFP methods.

In an initial evaluation, we considered energy (kcal/mol) as a performance metric, as it serves as the objective function of our hybrid optimization algorithm. In this context, lower energy values denote superior performance. This allowed a comparison between GRSA2-FCNN and the proposed GRSABio-FCNN, highlighting the performance enhancements achieved by GRSABio. However, since current state-of-the-art algorithms such as PEP-FOLD3, AlphaFold2, I-TASSER, Rosetta, and TopModel do not report energy values in their server outputs or results, comparisons with these methods were confined to structural metrics mentioned above, in Section 2.2.

### 2.1. Evaluation Between GRSA2-FCNN and GRSABio-FCNN

**Figure 1:**
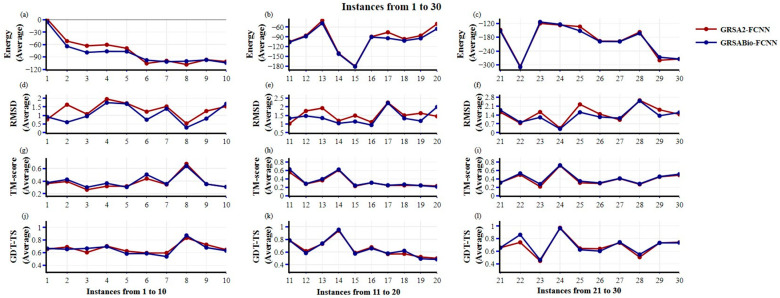
Comparison between GRSA2-FCNN and GRSABio-FCNN from 1 to 30 instances, using the metrics: Energy, RMSD, TM-score, and GDT-TS. (**a**–**c**) show the average of the five best predictions of Energy; (**d**–**f**) show the RMSD; (**g**–**i**) present the TM-score; and (**j**–**l**) show GDT-TS for each instance.

**Figure 2:**
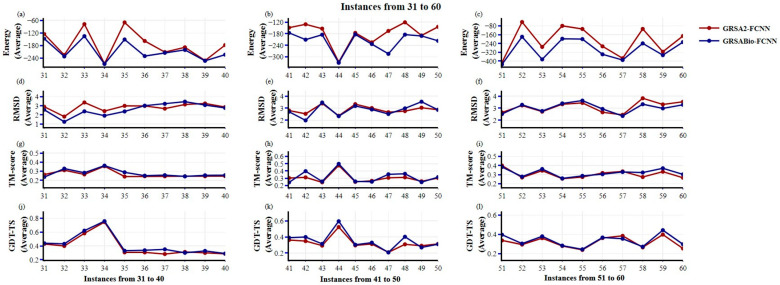
Comparison between GRSA2-FCNN and GRSABio-FCNN from 31 to 60 instances, using the metrics: Energy, RMSD, TM-score, and GDT-TS. (**a**–**c**) present the average of the five best predictions of Energy; (**d**–**f**) show the RMSD; (**g**–**i**) present the TM-score; and (**j**–**l**) show GDT-TS results.

The comparative analysis of GRSA2-FCNN and GRSABio-FCNN, as depicted in Figure 1 and Figure 2, is based on instances enumerated in Table 1, which includes a set of sixty peptides. The characteristics of each instance are also detailed in Table 1. For this evaluation, the instances are divided into two sets: the first set includes instances 1 to 30, corresponding to amino acid sequences with fewer than 30 residues; the second set includes instances 31 to 60, with sequences ranging from more than 30 to fewer than 50 amino acids. For both approaches (GRSA2-FCNN and GRSABio-FCNN), the average of the five best predictions was calculated, and their performance was compared across all instances. The first and second sets of result values for these approaches are presented in Table A1 and Table A2.

Regarding the energy metric in Figure 1, for instances 1 through 30, grouped into sets to highlight performance differences, GRSABio-FCNN demonstrates slightly better performance in instances 1 to 20 and similar performance in the last group. For the RMSD metric, where lower values (closer to zero) indicate higher structural quality, GRSABio-FCNN outperforms GRSA2-FCNN in the majority of instances. In terms of TM-score and GDT-TS metrics, where values closer to 1 signify better structural accuracy, GRSABio-FCNN shows superior performance in the first and third groups of ten instances for TM-score and consistently outperforms GRSA2-FCNN in instances 11 to 30 for GDT-TS, achieving the best scores in a greater number of instances.

For instances 31 to 60, grouped into sets of ten instances, as shown in Figure 2, GRSABio-FCNN significantly outperforms GRSA2-FCNN, demonstrating notable improvements in energy values across most instances, with consistently lower energy values. Regarding the RMSD metric, GRSABio-FCNN yields results comparable to those of GRSA2-FCNN, and in some cases, particularly for instances 31 to 50, even better. For the TM-score and GDT-TS, GRSABio-FCNN slightly outperforms GRSA2-FCNN across instances 31 to 60, achieving higher scores in several instances. Overall, in this second half of the dataset, GRSABio-FCNN exhibits enhanced performance in Energy, TM-score, and GDT-TS when compared to GRSA2-FCNN across the majority of instances.

Additionally, we compared GRSABio-FCNN and GRSA2-FCNN using the non-parametric Wilcoxon signed-rank test to assess statistically significant differences between their results. A 5% significance level was applied to determine whether the differences between the two algorithms were statistically meaningful. According to the test, a result is considered statistically significant if the calculated *p*-value is less than 0.05. Table 3 presents a summary of the test results. For example, in the energy results for peptides 1 to 30, GRSABio-FCNN performs better in 25 instances, while GRSA2-FCNN achieves better values in only 5 instances. GRSABio-FCNN outperforms GRSA2-FCNN in all the metrics for the entire dataset.

**Figure 3:**
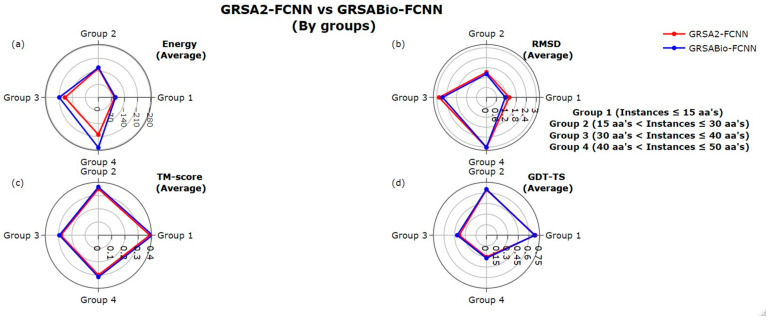
Comparison between GRSA2-FCNN and GRSABio-FCNN by groups, using the metrics: (**a**) Energy, (**b**) RMSD, (**c**) TM-score, and (**d**) GDT-TS.

Figure 3 illustrates the performance of GRSA2-FCNN and GRSABio-FCNN across four groups of peptide instances, each grouped by sequence length. The first group includes instances with up to 15 amino acids (aa), the second group includes instances between 16 and 30 aa, the third group includes instances between 31 and 40 aa, and the fourth group includes instances with more than 40 aa. For each group the average values corresponding to the peptide instances are presented. This analysis is applied to each evaluation metric: Energy, RMSD, TM-score, and GDT-TS.

In terms of Energy, GRSABio-FCNN shows in Figure 3a a notable improvement in groups 3 and 4 with reductions of −30 kcal/mol (13%) and −70 kcal/mol (35%), respectively. For RMSD, as illustrated in Figure 3b, GRSABio-FCNN slightly outperformed GRSA2-FCNN in groups 1, 2, and 3, while both models performed similarly in group 4. In the case of TM-score, GRSABio-FCNN achieved slightly improved performance across all four groups, as shown in Figure 3c. For GDT-TS in Figure 3d, GRSABio-FCNN also slightly outperformed GRSA2-FCNN in groups 3 and 4, whereas GRSA2-FCNN performed similarly in groups 1 and 2.

**Figure 4:**
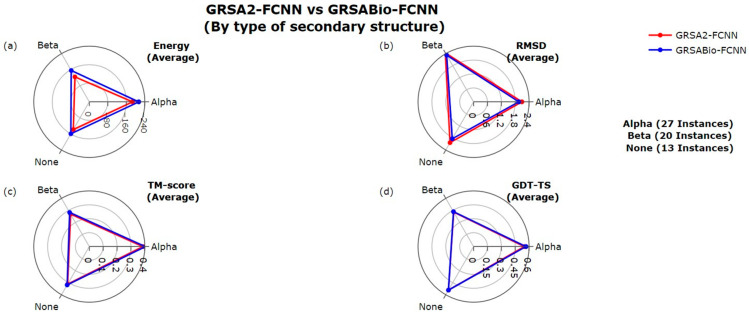
Comparison between GRSA2-FCNN and GRSABio-FCNN by type of secondary structure, using the metrics: (**a**) Energy, (**b**) RMSD, (**c**) TM-score, and (**d**) GDT-TS.

Figure 4 presents the results of GRSABio-FCNN and GRSA2-FCNN, categorized by secondary structures (SS) types, according to Table 1. The classification encompasses three groups: Alpha (28 instances), Beta (20 instances), and None (13 instances). The None type refers to instances lacking a majority of either alpha or beta structures. For each type of SS, a similar analysis was conducted using the evaluation metrics applied in the previous group comparisons. GRSABio-FCNN demonstrated superior performance over GRSA2-FCNN in terms of energy, with improvements of −26 kcal/mol (13%) for Alpha, −31 kcal/mol (18%) for Beta, and −20 kcal/mol (12%) for None, as shown in Figure 4a. For RMSD in Figure 4b, GRSABio-FCNN also achieved slightly better performance across all three SS types compared to GRSA2-FCNN. In terms of TM-score, GRSABio-FCNN showed slight improvements in the performance over GRSA2-FCNN for all SS types, as illustrated in Figure 4c. Finally, in Figure 4d, GRSABio-FCNN and GRSA2-FCNN demonstrated similar behaviors in the Alpha, Beta, and None types in GDT-TS. Overall, GRSABio-FCNN demonstrates superior performance compared to GRSA2-FCNN, particularly in energy, with modest gains in RMSD and TM-score.

### 2.2. Evaluation of GRSABio-FCNN and State-of-the-Art Algorithms

**Figure 5:**
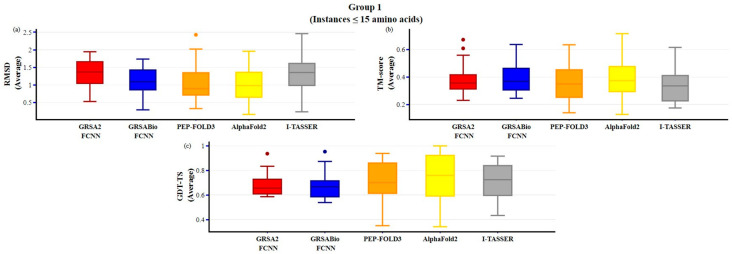
Comparison of GRSABio-FCNN with GRSA2-FCNN, PEP-FOLD3, AlphaFold2, and I-TASSER for instances with ≤15 amino acids, using the metrics: (**a**) RMSD, (**b**) TM-score, and (**c**) GDT-TS.

**Figure 6:**
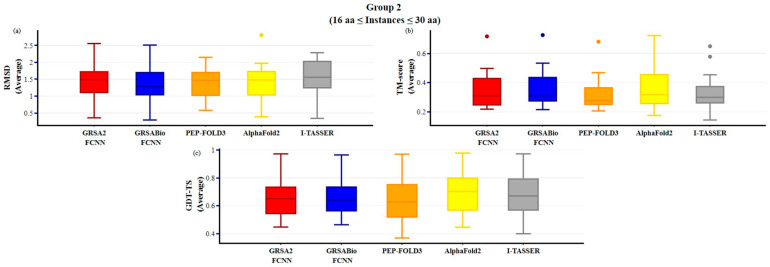
Comparison of GRSABio-FCNN with GRSA2-FCNN, PEP-FOLD3, AlphaFold2, and I-TASSER for instances ranging from 15 to 30 amino acids, using the metrics: (**a**) RMSD, (**b**) TM-score, and (**c**) GDT-TS.

**Figure 7:**
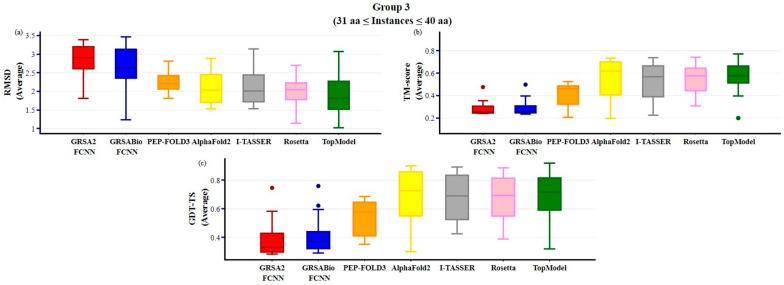
Comparison of GRSABio-FCNN with GRSA2-FCNN, PEP-FOLD3, AlphaFold2, I-TASSER, Rosetta, and TopModel for instances ranging from 31 to 40 amino acids, using the metrics: (**a**) RMSD, (**b**) TM-score, and (**c**) GDT-TS.

**Figure 8:**
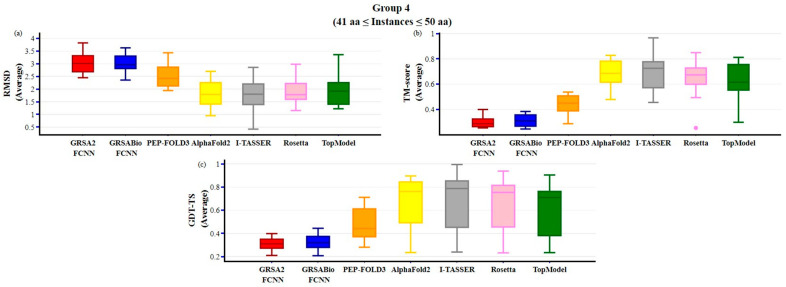
Comparison of GRSABio-FCNN with GRSA2-FCNN, PEP-FOLD3, AlphaFold2, I-TASSER, Rosetta, and TopModel for instances ranging from 41 to 50 amino acids, using the metrics: (**a**) RMSD, (**b**) TM-score, and (**c**) GDT-TS.

To assess the performance of GRSABio-FCNN, a comparative analysis was conducted against state-of-the-art approaches, PEP-FOLD3, AlphaFold2, I-TASSER, Rosetta, and TopModel, as presented in Figure 5, Figure 6, Figure 7 and Figure 8. Since these approaches do not report energy values in their outputs, the evaluation was limited to structural metrics: RMSD, TM-score, and GDT-TS. The dataset described in Table 1 was divided into four groups, each containing 15 instances, based on the number of amino acids in the peptide sequences: group 1 includes instances 1 to 15, group 2 includes instances from 16 to 30, group 3 includes instances from 31 to 45, and group 4 includes instances from 46 to 60. For each instance, the evaluation for each algorithm was based on the average of its five best predicted models. The results of this evaluation are presented in Table A3 and Table A4, in Appendix A. The state-of-the-art algorithms included in this evaluation are PEP-FOLD3, AlphaFold2, I-TASSER, GRSA2-FCNN, Rosetta, and TopModel. However, Rosetta and TopModel are not included in groups 1 and 2, as they generate predictions only for peptides and proteins with more than 27 and 30 amino acids, respectively.

In Figure 5 (Group 1), the performance of GRSABio-FCNN is evaluated in comparison with GRSA2-FCNN, PEP-FOLD3, AlphaFold2, and I-TASSER. In terms of RMSD, GRSABio-FCNN exhibits performance comparable to PEP-FOLD3 and AlphaFold2 and outperforms both GRSA2-FCNN and I-TASSER. For the TM-score, GRSABio-FCNN slightly outperforms PEP-FOLD3, GRSA2-FCNN, and I-TASSER, while showing comparable performance to AlphaFold2. However, when evaluating GDT-TS, AlphaFold2 demonstrates superior performance, achieving a maximum value of 1 and the highest median compared to the other algorithms.

In Table 4, GRSABio-FCNN is compared with state-of-the-art algorithms for Group 1 using the Wilcoxon signed-rank test. GRSABio-FCNN outperforms GRSA2-FCNN and I-TASSER in terms of RMSD. For the TM-score metric, GRSABio-FCNN surpasses all algorithms, achieving statistically significant improvement (at the 5% level) over I-TASSER. However, GRSABio-FCNN does not perform well in the GDT-TS metric.

In Figure 6, the results for group 2 are presented, where GRSABio-FCNN outperforms the other algorithms in terms of RMSD. GRSABio-FCNN and AlphaFold2 achieved similar results, both surpassing PEP-FOLD3, GRSA2-FCNN, and I-TASSER in the TM-score metric. AlphaFold2 demonstrates superior performance, with the highest median and a slightly better maximum value close to 1 for the GDT-TS metric.

In group 2, as depicted in Table 5, the GRSABio-FCNN algorithm demonstrates superior performance compared to GRSA2-FCNN, PEP-FOLD3, AlphaFold2, and I-TASSER in terms of RMSD, according to the Wilcoxon signed-rank test. Regarding the TM-score, GRSABio-FCNN exhibits statistically significant improvements over both GRSA2-FCNN and PEP-FOLD3. For the GDT-TS metric, GRSABio-FCNN outperforms PEP-FOLD3.

Figure 7 illustrates the results for group 3, which encompasses instances with peptide lengths exceeding 30 and up to 40 amino acids. GRSABio-FCNN outperforms GRSA2-FCNN across all three metrics: RMSD, TM-score, and GDT-TS. Overall, TopModel demonstrates superior performance in RMSD, achieving the lowest median, while AlphaFold2 excels in TM-score with the highest median value above 0.6. For GDT-TS, TopModel and AlphaFold2 exhibit similar performance, both reaching the highest value.

Table 6 presents the results for group 3 using the Wilcoxon signed-rank test. In this evaluation, GRSABio-FCNN outperforms GRSA2-FCNN in RMSD, TM-score, and GDT-TS, with statistical significance at the 5% level for the GDT-TS metric. Compared to the other algorithms, GRSABio-FCNN achieves approximately one-third of the positive cases against PEP-FOLD3 across RMSD, TM-score, and GDT-TS. Additionally, there are several instances (positive ranks) where GRSABio-FCNN performs better than the other algorithms, though it is not consistently the best across all structural metrics.

The results for group 4 are presented in Figure 8, with peptides of over 40 aa and up to 50 aa. In this group, GRSABio-FCNN demonstrates slightly better performance than GRSA2-FCNN across all the structural metrics. AlphaFold2, I-TASSER, and Rosetta achieved similar results, with the lowest median values in RMSD. For TM-score, I-TASSER outperformed the other algorithms, achieving the highest median value and demonstrating the best overall performance. Finally, I-TASSER also achieved the highest performance in the GDT-TS metric, with the best median value.

Table 7 presents the results for group 4 using the Wilcoxon signed-rank test. In this group, GRSABio-FCNN once again outperforms GRSA2-FCNN in RMSD, TM-score, and GDT-TS, with statistical significance at the 5% level for the GDT-TS metric. However, when compared to other algorithms in terms of structural metrics, GRSABio-FCNN does not achieve strong results. This is particularly evident in instances where the amino acid sequences are longer.

Furthermore, GRSABio-FCNN, along with the other state-of-the-art algorithms, was evaluated using the Friedman test on the dataset presented in Table 1. For this analysis, the dataset was divided into two groups: peptides 1 to 30 and peptides 31 to 60. The Friedman test ranks the algorithms based on their average performance, where a lower ranking score indicates better performance. The results are presented in Table 8 and Table 9. According to the statistical data presented in Table 4 and Table 5 for instances 1 to 30, GRSABio-FCNN achieves strong results in both RMSD and TM-score. However, as shown in Table 6 and Table 7, for instances 31 to 60, GRSABio-FCNN does not obtain the best rankings but still outperforms GRSA2-FCNN. AlphaFold2 achieves the highest rankings in the TM-score and GDT-TS metrics.

## 3. Discussion

In this section, we discuss the results obtained using the GRSABio-FCNN methodology. The complete dataset consists of 60 peptides, with a maximum number of 49 amino acids and up to 304 variables (torsion angles). As peptide length increases, the number of torsion variables grows significantly, leading to higher computational demands. The best results achieved by the methodology were observed in peptides with up to 30 amino acids, corresponding to a maximum number of 193 variables. These results demonstrate that GRSABio-FCNN explores the solution space more efficiently, maintaining high-quality structural prediction. In contrast, for longer peptides (over 30 aa), the increased complexity may hinder convergence to optimal solutions, thereby reducing overall prediction accuracy. Based on these results, the following aspects can be improved: One limitation is the use of in vacuo energy calculations, which consider only intramolecular interactions and neglect effects such as hydrophobic interactions and electrostatic screening. Another limitation lies in the design of the FCNN, which is trained to predict fragments of only six amino acids in length. This fixed fragment size is insufficient for accurately predicting longer peptides, particularly in instances involving extended sequences. As future work, we propose extending the fragment prediction to longer sequences and incorporating hyperparameter optimization for the Convolutional Neural Network and integrating solvent models or implicit solvation terms to better approximate the native folding environment.

## 4. Materials and Methods

### 4.1. Related Works on Protein Folding Problem (PFP)

PFP refers to the physical process by which a protein transitions from an unstable linear chain of amino acids into a more ordered three-dimensional structure, thereby becoming biologically functional [56]. To understand the complexity of this problem, several key points must be considered:

- The PFP is recognized as an NP-hard problem, indicating that the computational resources required to solve it increase exponentially with the size of the protein [11].
- Although a protein’s three-dimensional native structure is determined by its amino acid sequence, the vast conformational search space complicates accurate predictions.
- The Levinthal Paradox exemplifies this challenge: a protein cannot fold by randomly sampling all possible conformations due to the astronomical number of potential structures [57].

It is crucial to consider physical and chemical principles when addressing PFP, as the specific sequence of amino acids, or the primary structure of the protein, plays a pivotal role in determining this native state [58]. Various molecular forces, including hydrogen bonds, ionic interactions, and Van der Waals forces, significantly contribute to this process. Hydrogen bonds, which occur between the backbone elements and sometimes the side chains of the amino acids, help stabilize the helical and sheet structures that form early during folding. Ionic interactions, or salt bridges, form between positively and negatively charged side chains, further contributing to the stability of the protein’s folded state. Van der Waals forces, although individually weaker, collectively contribute to the overall compactness and stability of the folded protein by promoting tight packing of the protein’s interior [59]. These forces contribute to the potential energy used to score candidate structures to find the best conformation in the PFP.

One principle for obtaining the three-dimensional thermodynamically stable structure of a protein is based on the conformation with the lowest Gibbs-free energy [60]. This conformation is functional and represents the structure into which the protein naturally folds. One way to approximate the Gibbs-free energy is by estimating the internal potential energy of the protein. To determine the potential energy of a protein structure, it is essential to use force fields such as AMBER, CHARMM, OPLS, ECEPP/2, and ECEPP/3 [55,61,62]. In the case of ECEPP/2, the potential energy is given by Equation (1) and is calculated in vacuo, considering only intramolecular interactions [55]. In this work, ECEPP/2 serves as the objective function for determining and minimizing a protein structure’s energy.

(1) $$E_{total}=\sum_{j>i}\left(\frac{A_{ij}}{r_{ij}^{12}}-\frac{B_{ij}}{r_{ij}^{6}}\right)+332\sum_{j>i}\frac{q_{i}q_{j}}{\varepsilon r_{ij}}+\sum_{j>i}\left(\frac{C_{ij}}{r_{ij}^{12}}-\frac{D_{ij}}{r_{ij}^{10}}\right)+\sum_{n}U_{n}\left(1\pm cos\left(k_{n}\varphi_{n}\right)\right)$$

where *A_ij_*, *B_ij_*, *C_ij_*, and *D_ij_* represent the parameters of the empirical potentials; *r*_*ij*_ denotes the distance in angstroms (Å) between atoms *i* and *j*; *q_i_* and *q_j_* are their respective partial charges; *ε* is the dielectric constant; *U_n_* represents the energetic torsion barrier for rotation around bond *n*; and *K_n_* denotes the multiplicity of the torsion angle *φ_n_*.

#### 4.1.1. Computational Predictions for PFP

In the pursuit of accurate predictions of three-dimensional protein structures that closely resemble their native configurations, numerous methodologies have been developed in the literature. Notable examples include PEP-FOLD3 [43], AlphaFold2 [39], I-TASSER [50], Rosetta [40], and TopModel [51], all of which have demonstrated excellent results in predicting protein structures. Table 10 presents the main characteristics of these algorithms, along with their respective advantages, disadvantages, and constraints.

#### 4.1.2. Metaheuristic Algorithms for PFP

Metaheuristic algorithms have gained prominence in PFP due to their capacity to effectively explore complex and high-dimensional search spaces. These algorithms offer adaptable frameworks for optimizing solutions where conventional methods often prove inadequate, particularly in problems characterized by rugged energy landscapes and incomplete data [63]. Among these, Simulated Annealing (SA) is notable for its simplicity and effectiveness, as it probabilistically accepts suboptimal solutions to escape local optima, making it particularly suitable for refining protein structures or optimizing functional annotations. Concurrently, bio-inspired algorithms, which emulate natural processes, direct the search towards biologically plausible solutions. Their adaptability and global search capabilities have facilitated significant advancements in feature selection, motif detection, and protein classification [64]. Collectively, these metaheuristics serve as powerful tools that complement data-driven methods, contributing to deeper biological insights in PFP [15].

##### Simulated Annealing (SA) Algorithms

The SA algorithm is inspired by the annealing process in metallurgy, where metals are heated and gradually cooled to achieve a stable, low-energy state. Hybrid Simulated Annealing (HSA) algorithms are computational optimization techniques that enhance the principles of SA. Hybridization involves integrating additional methods, such as metaheuristics, deterministic algorithms, or machine learning techniques, to improve efficiency, accuracy, and robustness. This combination enables HSA to address complex optimization problems more effectively than classical SA.

A notable HSA algorithm for PFP is Golden Ratio Simulated Annealing (GRSA) [36], which enhances the cooling scheme in the classical SA algorithm by applying cuts at different temperature values calculated using the golden ratio (*φ*). This approach divides the cooling scheme into sections, where the temperature decreases based on a parameter (*α*) that varies within the range 0.7 ≤ *α* < 1 in each section. A value closer to one results in slower cooling and more extensive exploration of the solution space. The cooling scheme variation in GRSA depends on the number of cut-off temperatures (generated sections). To review the differences between GRSA and SA, we analyze their cooling schemes.

In classical SA, the number of iterations is determined by Equation (2), which does not account for cut-off temperatures:

(2) $$n_{SA-\alpha }=\frac{-C}{ln\left(\alpha \right)}$$

where *n_SA-α_* represents the number of iterations, and *C* is a constant determined by the final (*T_f_*) and initial (*T*_0_) temperature, given *C = LnT_f_ -LnT*_0_. The parameter *α* controls the rate of temperature decrement; the value of the decrement remains constant throughout the entire execution of the algorithm.

In GRSA, the temperature is divided into sections, typically up to a maximum of five [36], based on the golden ratio (*φ*). The initial temperature (*T*_0_) is multiplied by 0.618 (Φ), a value derived from the golden ratio (approximately 1.618), to determine the temperature of the next section (*T_GR_*), expressed as *T_GR_ =* 0.618 · *T*_0_. Unlike in SA, the number of iterations in GRSA increases with each new section because the parameter α is incremented according to α_new_ = α + 0.05. According to Equation (2), subsequent sections require more iterations than the first, resulting in a less intensive search in the later sections. This approach leads to less extensive exploration at high temperatures and more extensive exploration at lower temperatures in GRSA.

The GRSA algorithm reduces the execution time as the number of sections increases [36]. Experimental results presented in [36,41] indicate that utilizing five or fewer sections yields favorable outcomes for peptide prediction. Furthermore, in GRSA, a stopping criterion based on the least-squares method is employed to minimize exploration costs and reduce execution time in the final section. This criterion is determined by assessing the slope of the linear regression of the Metropolis cycle energy, as expressed in Equation (3):

(3) $$E_{i}=mi+b$$

where *E_i_* is the energy set for every *i*-metropolis cycle, *m* represents the slope, and *b* the interceptor.

Stochastic equilibrium is reached when *m* is close to zero, represented by Equation (4):

(4) $$m=\frac{k\sum_{i=2}^{k}iE_{i}-\left(\sum_{i=2}^{k}i\right)\left(\sum_{i=2}^{k}Ei\right)}{k\sum_{i=2}^{k}i^{2}-{\left(\sum_{i=2}^{k}i\right)}^{2}}$$

where *E_i_* represents the energy at each iteration, *k* is the number of Metropolis cycles, and *i* (iteration of every metropolis cycle) is within the range [2, *k_max_*], with *k_max_ =* 5 as in [36]. Equation (4) is then simplified into Equation (5):

(5) $$m=\frac{12\sum_{i=2}^{k_{max}}iE_{i}-6\left(k-1\right)\left(\sum_{i=2}^{k_{max}}Ei\right)}{k^{3}-k}$$

The GRSA, combined with another method, was evaluated on a set of peptides within the GRSA2-SSP [41] and GRSA2-FCNN [42] methodologies, demonstrating its effectiveness in comparison to state-of-the-art approaches.

#### 4.1.3. Bio-Inspired Algorithms

Bio-inspired algorithms are computational techniques that mimic natural processes and biological systems to solve complex optimization and problem-solving tasks. These algorithms provide heuristic and metaheuristic approaches that efficiently approximate near-optimal solutions [65]. Typically, bio-inspired algorithms follow four main steps: (1) Generate the Initial Population, (2) Apply Recombination Functions, (3) Evaluate the Objective Function and Constraints, and Selection, and (4) Compare Fitness and Update Best Vector for Next Generation. The overall process of a bio-inspired algorithm, along with the variables of the PFP, can be described as follows:

Generate the Initial Population. The initial step involves the random generation of a set of vectors, each comprising the variables pertinent to the problem. The number of variables defines the length of each vector, while the number of vectors establishes the size of the initial population. Each vector functions as a search agent, potentially representing entities such as animals (reptiles, mammals, amphibians, or insects), or even physical or chemical phenomena. The initial population undergoes evolution with each iteration. In the context of the PFP, the variables are the torsion angles that define the protein’s structure.

Apply Recombination Functions. In the subsequent step, a mathematical model is proposed to simulate the behavior of the modeled living entity, such as hunting, breeding, or mating. This model must achieve a balanced ratio between exploration (diversification) and exploitation (intensification) within the search solution space. In the context of the PFP, it generates new torsion angles, thereby creating new protein structures within the population.

Evaluate Objective Function and Constraints. In the third step, the set of vectors is evaluated using the objective function, with or without constraints. The outcome of this evaluation is referred to as fitness. When the objective is minimization, the vector with the lowest fitness value is considered the best. In the PFP context, the goal is to identify the structure with the lowest energy, corresponding to the best vector of torsion angles.

**Figure 9:**
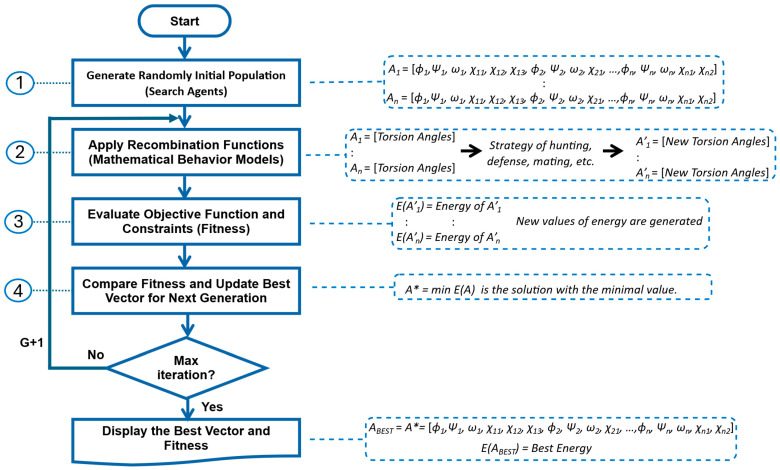
Generic flowchart of a bio-inspired algorithm applied to the PFP, with its corresponding variables evaluated through potential energy function.

Compare Fitness and Update Best Vector for Next Generation. In the final step, the best fitness value from the previous iteration (generation, G) is compared with the best fitness value from the current iteration. If the current value surpasses (in the case of minimization, is lower than) the best fitness obtained thus far, the value and its corresponding vector are updated. Consequently, in each iteration, the fitness will either remain the same (in the worst-case scenario) or improve, but it will not deteriorate. It is noteworthy that the performance of a bio-inspired algorithm can be significantly influenced by factors such as population size and the number of iterations. Figure 9 presents a generic flowchart of a bio-inspired algorithm, illustrating its application to the torsion angles in the PFP.

### 4.2. GRSABio-FCNN Methodology

**Figure 10:**
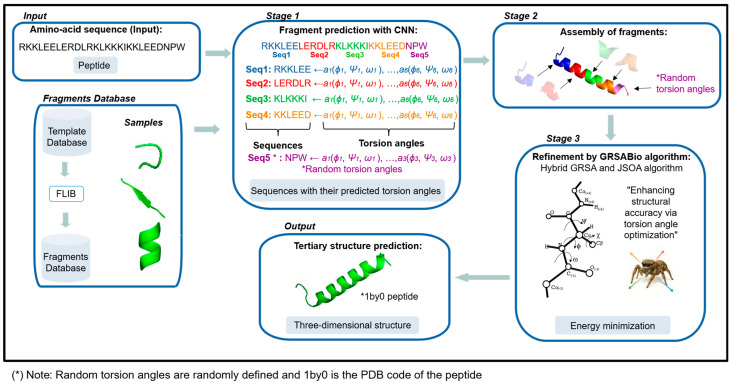
General scheme of GRSABio-FCNN methodology.

In this subsection, we introduce the GRSABio-FCNN methodology, enhanced with the Jumping Spider Optimization Algorithm (JSOA), to predict the three-dimensional structure of a peptide from its linear amino acid sequence. This enhanced methodology employs fragments generated from a Convolutional Neural Network (CNN), termed FCNN, to assemble an initial structure. Subsequently, this initial structure is refined using the bio-inspired JSOA until a three-dimensional conformation with minimal energy is attained. Figure 10 provides a general overview of the GRSABio-FCNN methodology. The input to this methodology is the linear amino acid sequence of the target protein. The stages of the methodology are described as follows:

- Amino Acid Sequence (Input) and Fragments Database. The amino acid sequence of the target protein, represented by a single-letter code, serves as the input for our method, while the fragments database contains a collection of fragments categorized based on their predominant secondary structures: alpha-helices, beta-sheets, and loops.
- Fragment Prediction with CNN (Stage 1). The fragment database serves as the input for training a CNN, which predicts fragments (alpha-helices, beta-sheets, and loops) along with their torsion angles—internal angles of the protein backbone, specifically phi (ϕ), psi (ψ), and omega (ω). The input amino acid sequence is segmented into short sequences, or fragments, each consisting of six amino acids, a length chosen to balance prediction accuracy while maintaining low computational requirements. The CNN processes these short sequences and generates their corresponding torsion angles for three-dimensional configuration.
- Assembly of Fragments (Stage 2). The predicted fragments, represented as vectors of torsion angles, are concatenated to construct a preliminary model of the target sequence. In other words, the individual predictions for each segment are combined sequentially to form a complete vector of torsion angles corresponding to the entire protein. During this process, the torsion angles of the fragments are assembled in segments of six amino acids based on the target sequence. If the size of the target sequence is not evenly divisible by the fragment size, resulting in missing angles for the final segment, random values are assigned to fill the gaps, which are refined in the next stage.
- Refinement by GRSABio Algorithm (Stage 3). The complete preliminary model, generated by concatenating the predicted fragments during the assembly phase, is refined using the GRSABio energy minimization process. This refined step optimizes the structure by reducing its energy, resulting in a more accurate and stable conformation.
- Tertiary structure prediction (Output). The outcome of refinement is the final tertiary structure of the target protein.

Figure 10 presents a general schematic of the GRSABio-FCNN, illustrating its stages along with the respective inputs and outputs.

In summary, GRSABio-FCNN begins with an amino acid sequence, which serves as input for a CNN trained on a fragment database. The CNN generates fragments based on the input sequence, and these fragments are assembled to form an initial protein structure. This preliminary structure is then refined using the novel hybrid GRSABio algorithm, resulting in a predicted tertiary structure. GRSABio-FCNN is an improved version of GRSA2-FCNN, akin to other methodologies in the literature that have been enhanced, such as PEP-FOLD3, I-TASSER, Rosetta, and AlphaFold. In Section 4.2.1, we provide a detailed explanation of the prediction and assembly of fragments. Additionally, Section 4.2.2 expands on the JSOA bio-inspired algorithm, offering a comprehensive overview of its principles and application.

#### 4.2.1. Prediction and Assembly Fragments

During the fragment prediction stage, a CNN is employed to predict fragments, a process referred to as FCNN. This prediction is based on a fragment library (Flib) [66] derived from known proteins in the Protein Data Bank (PDB) [52]. The library includes 12,368 alpha-like fragments, 9953 beta-like fragments, and 3576 loop-like fragments, each characterized by their amino acid sequences and torsion angles (ϕ, Ψ, and ω). These fragments serve as input for training the CNN. The CNN configuration and parameters from GRSA2-FCNN [42], which demonstrated high-quality fragment generation, were adopted and retained for GRSABio-FCNN to ensure consistent performance. The details are described below.

**Figure 11:**
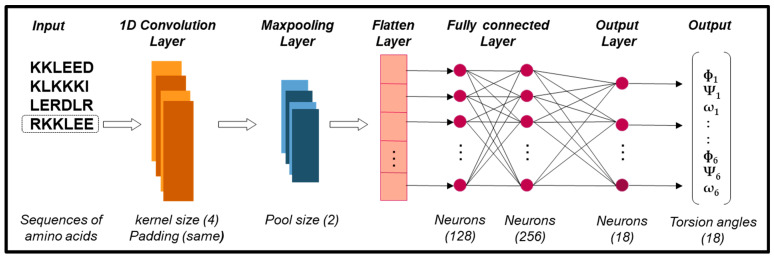
Architecture of FCNN.

The architecture of the CNN, shown in Figure 11, consists of four one-dimensional convolutional layers (1D CNN) with a kernel size of four, ReLU activation functions, and a dropout rate of 0.1, followed by a max-pooling layer with a size of two. The extracted features are flattened and processed through two fully connected layers with 128 and 256 neurons, before reaching an 18-neuron output layer. The network was trained using 80% of the dataset for training and 20% for validation, with an Adam optimizer [67], of which the mean square error (MSE) includes the loss function, a batch size of 8, and 200 epochs.

**Figure 12:**
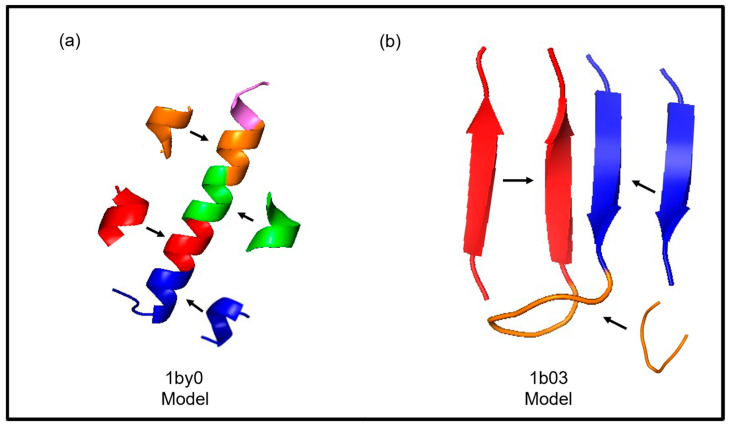
Examples of fragments generated by FCNN: 1by0 (**a**) and 1b03 (**b**).

In the assembly fragment stage, the new protein model is constructed by assembling short fragments predicted by the FCNN. Using the Flib database, FCNN predicts the torsion angles for the target sequence, concatenating fragments based on their amino acid positions. The resulting initial protein model, represented as Si = [ϕ_1_, Ψ_1_, Χ_1_, ω_1_, ϕ_2_, Ψ_2_, Χ_2_, ω_2_, …, ϕ_n_, Ψ_n_, Χ_n_, ω_n_], specifies the torsion angles for each amino acid. For instance, an instance with 32 amino acids is built using five fragments of six residues (amino acids) each, with the remaining amino acids initialized with random values by the GRSABio algorithm during refinement. Figure 12 illustrates two initial models generated by FCNN: peptide 1by0 (a) with a predominant alpha secondary structure and 1b03 (b) with a predominant beta secondary structure. These initial models are refined during the refinement stage.

#### 4.2.2. Refinement GRSABio

**Figure 13:**
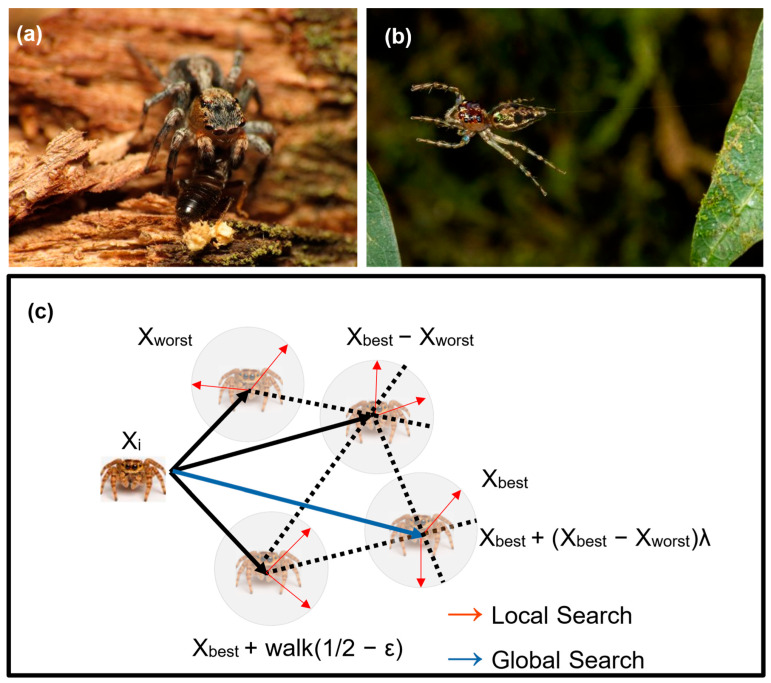
Representation of jumping spiders’ strategies. (**a**) Persecution strategy. Photography by Katja Schulz (published under a CC BY 2.0 license). (**b**) Jumping on the prey. Photography by Fresnelwiki (published under a CC BY-SA 4.0 license). (**c**) Local and global search.

GRSABio refines the initial model generated by the fragment assembly stage. This refinement integrates the GRSA algorithm with the JSOA to enhance the accuracy and quality of the final protein structure. The GRSA algorithm controls the exploration time, where the parameter alpha, used to decrement the temperature, ranges from 0.75 to 0.95 and is divided into five golden ratio sections. While GRSA controls the exploration time process, perturbations for generating new solutions from the initial solution vector S_i_ = [ ϕ _1_, Ψ_1_, Χ_1_, ω_1_, ϕ _2_, Ψ_2_, Χ_2_, ω_2_, …, ϕ_n_, Ψ_n_, Χ_n_, ω_n_] are generated by the JSOA, which evaluates these new solutions based on the minimization of the ECEPP/2 potential energy function. In the JSOA, various perturbation strategies are employed to explore the solution space, inspired by the behavioral patterns of the jumping spider from the Salticidae family [68]. This bio-inspired design leverages the characteristics of the jumping spider’s hunting strategies (persecution, search, and jumping). In addition to its hunting strategies, the spider’s pheromone range is considered [44]. The strategies of the jumping spider are illustrated in Figure 13.

The different strategies of the jumping spider are described below:

Persecution strategy. When the spider is too far to catch its prey by jumping, it approaches stealthily until within jumping range, as shown in Figure 13a. This strategy can be modeled using uniformly accelerated rectilinear motion, as shown in Equation (6), where the spider moves along a coordinate axis with its velocity increasing or decreasing linearly over time under constant acceleration:

(6) $$x_{i}=\frac{1}{2}at^{2}+v_{0}t$$

where *x_i_* represents the position of *i*-th follower spider, *t* denotes time, and *v*_0_ is the initial speed. The acceleration is calculated using *a = v/t*, where *v = x − x*_0._

Considering that Equation (6) must be applied for optimization using each iteration as a unit of time, and assuming that the difference between consecutive iterations is 1 and the initial velocity *v*_0_ *=* 0, Equation (7) can be derived as follows:

(7) $$x_{i}\left(g+1\right)=\frac{1}{2}({\vec{x}}_{i}\left(g\right)-{\vec{x}}_{r}(g))$$

Jumping on the prey strategy. The jumping spider tracks its prey and leaps to capture it, as seen in Figure 13b. This hunting behavior can be modeled as projectile motion, which combines uniform motion along the *X*-axis with uniformly accelerated motion along the *Y*-axis. The resulting trajectory of the spider’s leap can be described by Equation (8) as follows:

(8) $${\vec{x}}_{i}\left(g+1\right)={\vec{x}}_{i}\left(g\right)tan\left(\alpha \right)-\frac{{\vec{x}}_{i}^{2}\left(g\right)}{2V_{0}^{2}{cos}^{2}\left(\alpha \right)}\;\alpha =\frac{\phi \pi }{180}$$

where ${\vec{x}}_{i}$ (*g +* 1) represents the new position of a search agent, indicating the movement of jumping spiders. The $\vec{x_{i}}$ (*g*) is the current *i*-th search agent, *V*_0_ is set to 100 mm/seg, *g* is the gravitational acceleration (9.80665 m/s^2^), and the *α* angle is calculated using a randomly generated *ϕ* angle value within the interval (0,1).

Prey Searching Strategy. The jumping spider employs a random search within its environment to locate prey. This behavior is modeled using two mathematical functions: local and global search, as shown in Figure 13c. The local search is mathematically defined in Equation (9).

(9) $${\vec{x}}_{i}\left(g+1\right)={\vec{x}}_{best}\left(g\right)+walk\left(\frac{1}{2}-\varepsilon \right)$$

where ${\vec{x}}_{i}$ (*g +* 1) represents the new position of a search agent, while ${\vec{x}}_{best}$ (*g*) denotes the best search agent identified in the previous iteration. The term *walk* is a pseudo-random number uniformly distributed within the interval (−2,2), and ε is a pseudo-random number normally distributed within the range (0,1). Conversely, the global search is defined by Equation (10).

(10) $${\vec{x}}_{i}\left(g+1\right)={\vec{x}}_{best}\left(g\right)+\left({\vec{x}}_{best}\left(g\right)-{\vec{x}}_{worst}\left(g\right)\right)\lambda$$

where ${\vec{x}}_{i}$ (*g +* 1) represents the new position of a search agent, while ${\vec{x}}_{best}$ (*g*) and ${\vec{x}}_{worst}$ (*g*) correspond to the best and worst search agents in the previous iteration, respectively. Additionally, *λ* is a Cauchy random number with *μ* set to 0 and *θ* set to 1.

Jumping spider’s pheromone rates. Pheromones are chemical substances produced and secreted externally by an individual, which are detected through olfactory cues by other individuals of the same species, triggering behavioral changes. Many animals, including insects and spiders, produce pheromones. In some spider species, such as the black widow, courtship and mating behaviors are influenced not only by their striking coloration but also by pheromones. The modeling of pheromone production rates is based on [22] and is defined by the following Equation (11):

(11) $$pheromone\left(i\right)=\frac{{Fitness}_{max}-{Fitness}_{i}}{{Fitness}_{max}-{Fitness}_{min}}$$

where *Fitness_max_* and *Fitness_min_* are the worst and the best fitness value in the current generation, respectively, whereas *Fitness*(*i*) is the current fitness value of the *i*-th search agent. Equation (11) normalizes the fitness value in the interval (0,1) where 0 is the worst pheromone rate, whereas 1 is the best.

For low pheromone rate values (equal to or less than 0.3), Equation (12) is applied [22]:

(12) $${\vec{x}}_{i}\left(g\right)={\vec{x}}_{best}\left(g\right)+\frac{1}{2}\left({\vec{x}}_{r_{1}}\left(g\right)-{\left(-1\right)}^{\sigma }{\vec{x}}_{r_{2}}\left(g\right)\right)$$

where ${\vec{x}}_{i}$ (*g*) represents the search agent with a low pheromone rate; *r*_1_ and *r*_2_ are random integers generated within the interval [1, maximum size of search agents], with *r*_1_ *≠ r*_2_; ${\vec{x}}_{r_{1}}$ (*g*) and ${\vec{x}}_{r_{2}}$ (*g*) denote the *r*_1_-th and *r*_2_-th search agents, respectively; ${\vec{x}}_{best}$ (*g*) is the best search agent found in the previous iteration; *σ* is a binary number randomly generated, where *σ* ϵ {0,1}. The persecution, jumping on the prey, prey searching strategies, and pheromone rate of the jumping spider represented by their respective equations, are implemented within the JSOA. These strategies are then integrated into the GRSA algorithm to create a more effective hybrid optimization algorithm.

The procedures for GRSA, including the cooling scheme and stopping criterion, are incorporated in Algorithm 1, while JSOA, along with its strategies and pheromone updating process, is detailed in Algorithms 2 and 3, respectively.

In the pseudocode of Algorithm 1, GRSABio, the parameters Ti and Tf define the initial and final temperatures, respectively. The parameters *α* and Φ represent the cooling factor and the golden number, respectively. The initial solution contains the torsion angles of the protein structure and is provided by the predictions of the CNN-predicted fragments. In line 5, the algorithm initiates the temperature cycle, bounded by Ti and Tf. The Metropolis cycle, implemented in line 6, generates new solutions using the BioperturbationJSOA function based on JSOA strategies. The Metropolis length is denoted by L_k_ [69], representing the length of the Markov chain during the kth temperature cycle. This parameter represents the number of times the solution space is explored at a fixed temperature and is defined as the number of iterations performed within the Metropolis cycle for the kth cycle [47,69]. The energy of each candidate solution (fitness) is obtained with the ECEPP/2 potential energy function. The acceptance or rejection of new solutions is determined by the conditions in lines 9 and 12, following an acceptance criterion based on the Boltzmann distribution (lines 12–14). The algorithm continues iterating until the actual temperature Tk reaches the initial temperature Ti. The analytical tuning method applied in this work for GRSA can be found in [47].
**Algorithm 1** GRSABio Algorithm
1:     Data: Tf, Tfp, Ti, E, S, *α* 2:     α = 0.70; Φ = 0.618 3:     Tfp = Ti; Tk = Ti 4:     Si = InitialSolution() 5:     **while** Tk ≥ Tf do //*Temperature cycle* 6:                        **while** Metropolis length do //*Metropolis cycle* 7:                  Sj = BioperturbationJSOA(Si) 8:                  ΔE = Energy(Sj) − Energy(Si) 9:            **if** ΔE ≤ 0 **then** 10:                                  Si = Sj 11:                                  E = Energy(Si) 12:          **else if** e^−ΔE/Ti^ < random [0-1] **then**
13:                                  Si = Sj 14:                                  E = Energy(Si) 15:          **end if** 16:                  **end while** //*End Metropolis cycle* 17:                  GRSA_Cooling_Schema(Tfp) 18:                  GRSA_Stop_Criterion() 19:     **end while** //*End Temperature cycle*

In the BioperturbationJSOA function (Algorithm 2), JSOA strategies are applied to randomly generated agents based on the previous solution *Si* (line 1). These strategies are executed within a loop controlled by the maximum number of iterations, during which different behavioral strategies are applied. The attack strategies, persecution, and jumping on the prey, are implemented in lines 7 and 9, while the search strategies, including local and global search, are in lines 13 and 15, respectively. In line 18, the pheromone update procedure is applied to determine the best energy (fitness value) within the population, using the ECEEP/2 force field to calculate the energy. Subsequently, in lines 19 to 21, the fitness values are evaluated. The function terminates once the maximum number of iterations is reached. The best solution obtained from the BioperturbationJSOA, representing the optimal conformation of torsion angles, is returned to the Metropolis cycle in Algorithm 1.
**Algorithm 2** BiopertubationJSOA Function
1:     BioperturbationJSOA(Si) 2:     n=MaxIteration 3:     Agents = InitialAgents() 4:     **while** iteration < n **do**
5:                           **if** random < 0.5 **then** Attack or Search? 6:                                            **if** random < 0.5 **then** Strategy 1 7:                Attack by persecution, Equation (7) 8:                **else** Strategy 2 9:                Attack by jumping on the prey, Equation (8) 10:                                        **end if** 11:                      **else** 12:              **if** random < 0.5 **then** Strategy 3 Local Search 13:              Search for prey by local search, Equation (9) 14:              **else** Strategy 3 Global Search 15:              Search for prey by global search, Equation (10) 16:              **end if** 17:                    **end if** 18:                      Update search agents with pheromone by Equations (11) and (12) 19:                      bestSolution = BestAgent(Si) 20:                      Iteration = Iteration + 1 21:    **end while**
22:    **end** Function

The pheromone procedure in Algorithm 3 is executed utilizing the pheromone rate defined in Equation (11) and the criterion specified in Equation (12), as implemented in lines 2 and 5, respectively.
**Algorithm 3** Pheromone procedure
1:      Pheromone procedure 2:      Compute *pheromone* rate for all spiders (search agents) by Equation (11)) 3:      **for** i = 1 to sizePopulation **do**
4:                           **if** *pheromone*(i) ≤ 0.3 **then** 5:                                     search agent update by Equation (12) 6:                            **end if** 7:      **end for**
8:      return $\vec{x}$

9:      **end** procedure

**Figure 14:**
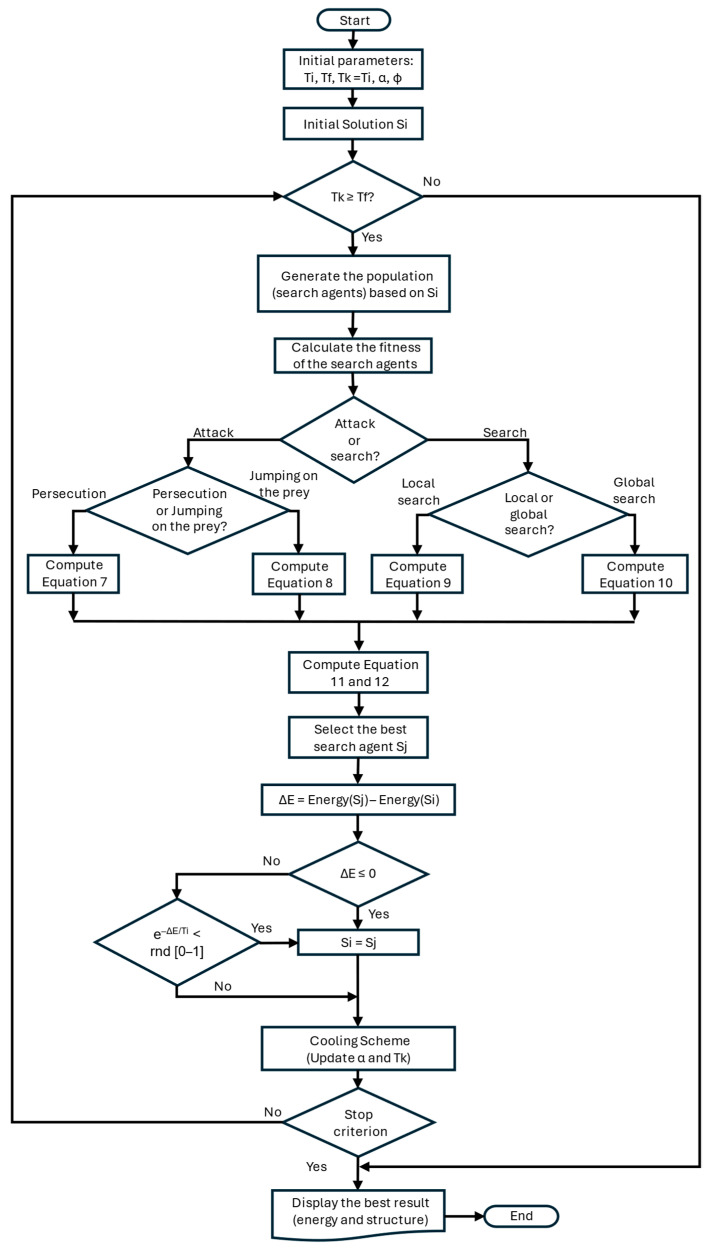
Flowchart of GRSABio.

The flowchart in Figure 14 illustrates the integration of the main features of the simulated annealing and jumping spider optimization algorithms. It begins with an initial solution at a high temperature, iteratively generates new solutions in each cycle. A solution is accepted based on an acceptance criterion that considers the difference between the new and previous solutions. As the temperature decreases through the cooling scheme, it becomes increasingly difficult to accept worse solutions, guiding the search toward optimal regions. The algorithm includes a stop criterion to terminate the process. The golden ratio is incorporated to enhance exploration efficiency across different sections of the cooling scheme, while the JSOA is employed to explore the solution space using different bio-inspired strategies aimed at obtaining better solutions.

**Figure 15:**
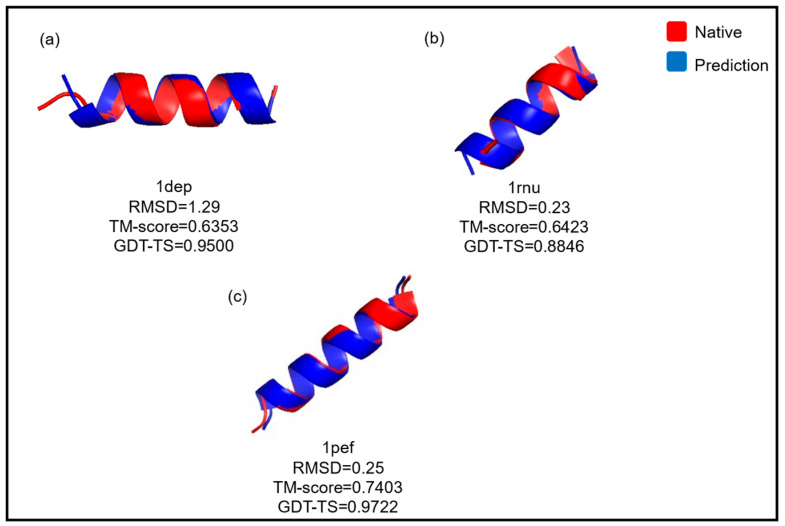
Three-dimensional models of peptides refined by GRSABio (blue) and their corresponding native structure (red). Subfigures (**a**–**c**) illustrate the superposition of the native and predicted structures for the peptides 1dep, 1rnu, and 1pef, respectively.

Figure 15 illustrates the refinement of three models obtained by the GRSABio algorithm, alongside the native structure, evaluated using the TM-score and GDT-TS metrics [70].

Computational Complexity of GRSABio Refinement

GRSABio is a hybrid integration of the GRSA and JSOA, both designed to solve optimization problems, which involve computational time complexity. In the case of SA, the complexity is $(n^{2}+n)\log n$, where n corresponds to the number of variables in each peptide [71]. For GRSA, the number of iterations is equal to or fewer than those in SA and is associated with the number of iterations required to generate new solutions; therefore, GRSA maintains the same complexity as SA [47]. The computational time complexity of JSOA is defined as O(f(n) ∗ tMax ∗ nSpiders), where f(n) represents the energy function presented in Equation (1), tMax is the number of iterations and nSpiders is the population size [44]. Based on the complexities of GRSA and JSOA, the overall computational complexity of GRSABio is defined as $O(\left(\left(n^{2}+n\right)\log n\right)\;*\;f\left(n\right)*tMax\;*\;nSpiders)$.

## 5. Conclusions

This work introduces a novel hybrid optimization algorithm, termed GRSABio, which integrates Golden Ratio Simulated Annealing (GRSA) with the Bio-inpired Jumping Spider Optimization Algorithm (JSOA). This hybrid approach has been incorporated into the enhanced prediction methodology, GRSABio-FCNN. The proposed algorithm leverages the strategies of the JSOA, including search, persecution, and jumping on the prey, integrated with the cooling scheme of GRSA to enhance the efficiency of exploring the solution space.

The GRSABio-FCNN methodology was evaluated using a dataset of 60 peptides, with performance analysis conducted on individual peptides, grouped by sequence length, and by secondary structure type. GRSABio-FCNN was benchmarked against several state-of-the-art algorithms, including GRSA2-FCNN, PEP-FOLD3, AlphaFold2, I-TASSER, Rosetta, and TopModel. According to the energy results, GRSABio-FCNN surpasses GRSA2-FCNN on peptides prediction, achieving significantly lower energy values and yielding superior solutions. Notably, only these two approaches provide energy outputs. These findings are corroborated by the Wilcoxon signed-rank test and the Friedman test. For peptides with up to 30 aa, as evaluated using structural metrics, GRSABio-FCNN demonstrates competitive performance compared to current state-of-the-art methods, often achieving good or superior results in many cases of peptides. However, its performance on longer peptide sequences (above 30 aa) is competitive in some cases, while less favorable in others.

The proposed enhanced GRSABio-FCNN achieves exceptional results for peptides up to 30 aa in terms of structural metrics, and remains competitive with leading algorithms in the field, underscoring its potential as an effective tool for protein structure prediction. Future research will focus on incorporating additional force fields and adjusting fragment sizes to better accommodate larger peptides.

## Figures and Tables

**Table 1:** Dataset of peptides [42].

Method	Type SS	Variables	Aa	PDB-Code	No	Method	Type SS	Variables	Aa	PDB-Code	No
NMR	N	163	31	1t0c	31	NMR	N	49	9	1egs	1
NMR	A	201	31	2gdl	32	NMR	B	47	10	1uao	2
NMR	A	183	32	2l0g	33	NMR	N	62	12	1l3q	3
NMR	A	200	33	2bn6	34	NMR	B	66	12	2evq	4
NMR	A	210	34	2kya	35	NMR	B	69	12	1le1	5
NMR	B	197	36	1wr3	36	NMR	A	74	12	1in3	6
NMR	B	206	36	1wr4	37	X-ray	N	61	13	1eg4	7
NMR	B	206	37	1e0m	38	X-ray	A	81	13	1rnu	8
NMR	B	212	37	1yiu	39	NMR	N	81	13	1lcx	9
NMR	B	221	37	1e0l	40	X-ray	N	74	14	3bu3	10
NMR	N	216	38	1bhi	41	NMR	A	79	14	1gjf	11
NMR	B	208	39	1jrj	42	NMR	B	84	14	1k43	12
NMR	A	218	39	1i6c	43	NMR	N	85	14	1a13	13
NMR	A	242	39	1bwx	44	NMR	A	94	15	1dep	14
NMR	B	213	40	2ysh	45	NMR	N	100	15	2bta	15
NMR	B	222	41	1wr7	46	NMR	A	86	16	1nkf	16
NMR	A	279	41	1k1v	47	NMR	B	91	16	1le3	17
NMR	A	268	42	2hep	48	X-ray	B	93	16	1pgbF	18
NMR	A	229	43	2dmv	49	NMR	B	97	16	1niz	19
NMR	B	268	43	1res	50	NMR	B	109	17	1e0q	20
NMR	A	295	44	2p81	51	NMR	N	120	17	1wbr	21
NMR	B	247	45	1ed7	52	NMR	A	124	17	1rpv	22
NMR	A	276	45	1f4i	53	NMR	B	109	18	1b03	23
NMR	B	250	46	2l4j	54	X-ray	A	124	18	1pef	24
NMR	A	272	47	1qhk	55	NMR	A	100	20	1l2y	25
NMR	A	279	47	1dv0	56	NMR	A	134	20	1du1	26
NMR	N	304	47	1pgy	57	NMR	A	143	22	1pei	27
NMR	N	294	48	1e0g	58	NMR	A	123	23	1wz4	28
NMR	N	290	49	1ify	59	NMR	A	160	27	1yyb	29
NMR	A	303	49	1nd9	60	NMR	A	193	27	1by0	30

Note: The rows in the table are sorted by the number of amino acids (aa), its type of structure secondary (SS) in terms of alpha-helical (A), beta-sheet (B), and none (N).

**Table 2:** Parameter setting of algorithms.

Approach	Parameter	Typical Value/Description
GRSA2-FCNN	A	[0.70, 0.95]
	Φ	0.618
	Fragment length (residues)	6
GRSABio-FCNN	A	[0.70, 0.95]
	Φ	0.618
	Fragment length (residues)	6
	Number of agents	10
	Maximum Iterations	20
PEP-FOLD3	Number of simulations	100
	Fragment library	Precomputed structural motifs from known peptides
AlphaFold2	Number of recycles	3
	MSA * depth	~512
	Structure module iterations	Typically, 3–8
	Model confidence score	pLDDT (0–100)
I-TASSER	Number of threading templates	Top 10 from LOMETS
	Number of Monte Carlo simulations	20 models
	Clustering method	SPICKER
Rosetta	Fragment length (residues)	(3–9)
	Number of decoys	(1000–10,000)
	Energy function	Rosetta score12
TopModel	Scoring model	Deep neural network scoring

* MSA refers to multiple sequence alignment. Parameter “A” refers to alpha (α).

**Table 3:** Statistical results of Wilcoxon signed-rank test for GRSABio-FCNN versus GRSA2-FCNN for dataset, with a significance level of 5%.

Instances	Energy GRSABio-FCNN vs. GRSA2-FCNN	RMSD GRSABio-FCNN vs. GRSA2-FCNN	TM-score GRSABio-FCNN vs. GRSA2-FCNN	GDT-TS GRSABio-FCNN vs. GRSA2-FCNN
From 1 to 30	(+/=/−) 25/0/5 *p*-value: **5.00 × 10^−3^**	(+/=/−) 22/0/8 *p*-value: **8.73 × 10^−3^**	(+/=/−) 25/1/4 *p*-value: **4.41 × 10^−4^**	(+/=/−) 11/1/18 *p*-value: 4.96 × 10^−1^
From 31 to 60	(+/=/−) 30/0/0 *p*-value: **2.00 × 10^−6^**	(+/=/−) 19/0/11 *p*-value: 7.86 × 10^−2^	(+/=/−) 22/0/8 *p*-value: **2.06 × 10^−2^**	(+/=/−) 25/0/5 *p*-value: **8.73 × 10^−3^**
From 1 to 60	(+/=/−) 55/0/5 *p*-value: **6.31 × 10^−9^**	(+/=/−) 41/0/19 *p*-value: **2.60 × 10^−3^**	(+/=/−) 47/1/12 *p*-value: **4.20 × 10^−5^**	(+/=/−) 36/1/23 *p*-value: **3.86 × 10^−2^**

Note: The bold numbers in the table indicate a significant difference between the GRSA2-FCNN and GRSABio-FCNN, where GRSABio was outstanding.

**Table 4:** Statistical results of Wilcoxon signed-rank test for group 1, with a significance level of 5%.

Algorithms	RMSD	TM-Score	GDT-TS
GRSABio-FCNN vs. GRSA2-FCNN	(+/=/−) 12/0/3 *p*-value: **1.67 × 10^−2^**	(+/=/−) 12/1/2 *p*-value: **1.85x10^−2^**	(+/=/−) 5/0/10 *p*-value: 2.80x10^−1^
GRSABio-FCNN vs. PEP-FOLD3	(+/=/−) 6/0/9 *p*-value: 9.54 × 10^−1^	(+/=/−) 11/0/4 *p*-value: 9.95 × 10^−2^	(+/=/−) 5/0/10 *p*-value: **7.82 × 10^−2^**
GRSABio-FCNN vs. AlphaFold2	(+/=/−) 8/0/7 *p*-value: 7.76 × 10^−1^	(+/=/−) 10/0/5 *p*-value: 3.34 × 10^−1^	(+/=/−) 4/0/11 *p*-value: 1.91 × 10^−1^
GRSABio-FCNN vs. I-TASSER *	(+/=/−) 9/0/5 *p*-value: 2.71 × 10^−1^	(+/=/−) 12/0/2 *p*-value: **9.18 × 10^−3^**	(+/=/−) 5/0/9 *p*-value: 4.70 × 10^−1^

* I-TASSER provides results for only 14 instances due to its limitation in predicting sequences shorter than 15 amino acids. The bold numbers in the table indicate a significant difference between algorithms, with the algorithm that has the most “+” being the most outstanding.

**Table 5:** Statistical results of Wilcoxon signed-rank test for group 2, with a significance level of 5%.

Algorithms	RMSD	TM-Score	GDT-TS
GRSABio-FCNN vs. GRSA2-FCNN	(+/=/−) 10/0/5 *p*-value: 1.72 × 10^−1^	(+/=/−) 13/0/2 *p*-value: **6.39 × 10^−3^**	(+/=/−) 6/0/8 *p*-value: 9.24 × 10^−1^
GRSABio-FCNN vs. PEP-FOLD3	(+/=/−) 9/0/6 *p*-value: 6.90 × 10^−1^	(+/=/−) 13/0/2 *p*-value: **1.70 × 10^−2^**	(+/=/−) 10/0/5 *p*-value: 3.06 × 10^−1^
GRSABio-FCNN vs. AlphaFold2	(+/=/−) 9/0/6 *p*-value: 6.49 × 10^−1^	(+/=/−) 10/0/5 *p*-value: 6.90 × 10^−1^	(+/=/−) 4/0/11 *p*-value: 9.95 × 10^−2^
GRSABio-FCNN vs. I-TASSER	(+/=/−) 11/0/4 *p*-value: 3.63 × 10^−1^	(+/=/−) 11/0/4 *p*-value: 1.91 × 10^−1^	(+/=/−) 5/0/10 *p*-value: 5.70 × 10^−1^

The bold numbers in the table indicate a significant difference between algorithms, with the algorithm that has the most “+” being the most outstanding.

**Table 6:** Statistical results of Wilcoxon signed-rank test for group 3, with a significance level of 5%.

Algorithms	RMSD	TM-Score	GDT-TS
GRSABio-FCNN vs. GRSA2-FCNN	(+/=/−) 11/0/4 *p*-value: **4.08 × 10^−2^**	(+/=/−) 12/0/3 *p*-value: 6.91 × 10^−2^	(+/=/−) 14/0/1 *p*-value: **1.46 × 10^−3^**
GRSABio-FCNN vs. PEP-FOLD3	(+/=/−) 5/0/10 *p*-value: **3.56 × 10^−2^**	(+/=/−) 4/0/11 *p*-value: **7.59 × 10^−3^**	(+/=/−) 5/0/10 *p*-value: 6.08 × 10^−2^
GRSABio-FCNN vs. AlphaFold2	(+/=/−) 4/0/11 *p*-value: **3.08 × 10^−2^**	(+/=/−) 4/0/11 *p*-value: **4.51 × 10^−3^**	(+/=/−) 3/0/12 *p*-value: **6.40 × 10^−3^**
GRSABio-FCNN vs. I-TASSER	(+/=/−) 5/0/10 *p*-value: **3.56 × 10^−2^**	(+/=/−) 2/0/13 *p*-value: **8.05 × 10^−4^**	(+/=/−) 1/0/14 *p*-value: **3.14 × 10^−3^**
GRSABio-FCNN vs. Rosetta	(+/=/−) 3/0/12 *p*-value: **1.05 × 10^−2^**	(+/=/−) 1/0/14 *p*-value: **8.05 × 10^−4^**	(+/=/−) 2/0/13 *p*-value: **3.77 × 10^−3^**
GRSABio-FCNN vs. Top-Model	(+/=/−) 3/0/12 *p*-value: **4.92 × 10^−3^**	(+/=/−) 2/0/13 *p*-value: **1.20 × 10^−3^**	(+/=/−) 3/0/12 *p*-value: **4.51 × 10^−3^**

The bold numbers in the table indicate a significant difference between algorithms, with the algorithm that has the most “+” being the most outstanding.

**Table 7:** Statistical results of Wilcoxon signed-rank test for group 4, with a significance level of 5%.

Algorithms	RMSD	TM-Score	GDT-TS
GRSABio-FCNN vs. GRSA2-FCNN	(+/=/−) 8/0/7 *p*-value: 7.76 × 10^−1^	(+/=/−) 8/0/7 *p*-value: 3.06 × 10^−1^	(+/=/−) 11/0/4 *p*-value: **4.68 × 10^−2^**
GRSABio-FCNN vs. PEP-FOLD3	(+/=/−) 2/0/13 *p*-value: **1.78 × 10^−3^**	(+/=/−) 5/0/10 *p*-value: 1.39 × 10^−1^	(+/=/−) 2/0/13 *p*-value: **3.77 × 10^−3^**
GRSABio-FCNN vs. AlphaFold2	(+/=/−) 1/0/14 *p*-value: **8.05 × 10^−4^**	(+/=/−) 2/0/13 *p*-value: **1.20 × 10^−3^**	(+/=/−) 2/0/13 *p*-value: **1.20 × 10^−3^**
GRSABio-FCNN vs. I-TASSER	(+/=/−) 0/0/15 *p*-value: **6.53 × 10^−4^**	(+/=/−) 2/0/13 *p*-value: **1.47 × 10^−3^**	(+/=/−) 2/0/13 *p*-value: **1.20 × 10^−3^**
GRSABio-FCNN vs. Rosetta	(+/=/−) 1/0/14 *p*-value: **8.05 × 10^−4^**	(+/=/−) 2/0/13 *p*-value: **4.51 × 10^−3^**	(+/=/−) 2/0/13 *p*-value: **1.20 × 10^−3^**
GRSABio-FCNN vs. TopModel	(+/=/−) 2/0/13 *p*-value: **1.47 × 10^−3^**	(+/=/−) 2/0/13 *p*-value: **2.61 × 10^−3^**	(+/=/−) 2/0/13 *p*-value: **1.47 × 10^−3^**

The bold numbers in the table indicate a significant difference between algorithms, with the algorithm that has the most “+” being the most outstanding.

**Table 8:** Friedman test results for all compared algorithms based on structural metrics for instances 1 to 30.

Algorithms	RMSD		TM-Score		GDT-TS	
	Mean of Ranks	Overall of Ranks	Mean of Ranks	Overall of Ranks	Mean of Ranks	Overall of Ranks
GRSA2-FCNN	3.48	**4**	2.95	**3**	3.34	**4**
GRSABio-FCNN	2.45	**1**	1.91	**1**	3.53	**5**
PEP-FOLD3	2.59	**2**	3.55	**4**	3.22	**3**
AlphaFold2	2.97	**3**	2.76	**2**	2.07	**1**
I-TASSER	3.52	**5**	3.83	**5**	2.83	**2**

The bold number in the table indicates the order of the ranked algorithms by the Friedman test.

**Table 9:** Friedman test results for all compared algorithms based on structural metrics for instances 31 to 60.

Algorithms	RMSD		TM-Score		GDT-TS	
	Mean of Ranks	Overall of Ranks	Mean of Ranks	Overall of Ranks	Mean of Ranks	Overall of Ranks
GRSA2-FCNN	6.32	**7**	6.47	**7**	6.33	**7**
GRSABio-FCNN	5.50	**6**	5.77	**6**	5.37	**6**
PEP-FOLD3	4.52	**5**	5.03	**5**	4.98	**5**
AlphaFold2	2.87	**3**	2.28	**1**	2.37	**1**
I-TASSER	3.30	**4**	2.53	**2**	2.65	**2**
Rosetta	2.72	**1**	2.77	**3**	3.02	**3**
TopModel	2.78	**2**	3.15	**4**	3.28	**4**

The bold number in the table indicates the order of the ranked algorithms by the Friedman test.

**Table 10:** State-of-the-art algorithmic approaches in PFP.

Approach	Features	Advantages	Disadvantages	Constraints
**PEP-FOLD3**	Specialized in de novo prediction of short peptides (up to 50 amino acids).	Fast and easy to use; good for small peptides	Not suitable for large proteins; limited structural accuracy for complex folds.	Limited to peptides; does not handle large or multi-domain proteins.
**AlphaFold2**	Deep learning-based, uses evolutionary, structural, and physical data.	State-of-the-art accuracy; predicts full atom-level protein structures.	Computationally intensive; model architecture is complex.	Requires multiple sequence alignment (MSA) and significant computing resources; not ideal for short peptides.
**I-TASSER**	Threading-based with ab initio modeling; ranks models using clustering (from 10 to 1500 amino acids).	Good for proteins with known homologs; provides function prediction.	Less accurate for proteins without templates; longer computation times.	Dependent on structural templates; less effective for novel folds.
**Rosetta**	Uses fragment assembly and energy minimization; highly customizable suitable for sequences starting from 27 amino acids	Versatile for structure, docking, and design; proven across many scenarios.	High complexity; steep learning curve; requires fine-tuning.	Demands significant CPU/GPU time and technical knowledge to set up properly
**TopModel**	Combines deep learning with consensus scoring; designed for model quality assessment, applicable from sequences as short as 30 amino acids.	Enhances reliability of predicted structures by model quality evaluation.	Not a structure predictor itself; relies on input from other predictors.	Works as a complementary tool; does not generate initial models.

Bold text in the table denotes the commonly recognized names of the respective approaches.

## Data Availability

Publicly available datasets were analyzed in this study and source code used. This data and source code used can be found here: https://github.com/DiegoSoto87/ResultsGRSABioFCNN.git (accessed on 28 July 2025) and https://github.com/DiegoSoto87/GRSABio-FCNN.git (accessed on 28 July 2025).

## Appendix A

**Table A1:** Result values of GRSA2-FCNN and GRSABio-FCNN from 1 to 30 instances, using the metrics: Energy, RMSD, TM-score, and GDT-TS.

	Energy Ave.		RMSD Ave.		TM-Score Ave.		GDT-TS Ave.	
Instances	GRSA2-FCNN	GRSABio-FCNN	GRSA2-FCNN	GRSABio-FCNN	GRSA2-FCNN	GRSABio-FCNN	GRSA2-FCNN	GRSABio-FCNN
1. 1egs	1.6866	**−5.7061**	**0.7620**	0.9120	0.3615	**0.3744**	0.6556	**0.6666**
2. 1uao	−51.7199	**−64.1566**	1.6200	**0.6020**	0.3932	**0.4241**	**0.6900**	0.6550
3. 1l3q	−63.0239	**−79.0836**	1.0740	**0.9500**	0.2629	**03013**	0.6041	**0.6667**
4. 2evq	−60.5334	**−76.3770**	1.9440	**1.7360**	0.3182	**0.3646**	**0.7042**	0.6958
5. 1le1	−69.0947	**−76.8187**	1.7020	**1.6680**	**0.3179**	0.3047	**0.6250**	0.5834
6. 1in3	**−105.5510**	−97.7497	1.2140	**0.7480**	0.4393	**0.5034**	**0.5958**	0.5875
7. 1eg4	−99.2259	**−101.5050**	1.5160	**1.3840**	0.3456	**0.3545**	**0.5962**	0.5385
8. 1rnu	**−108.2189**	−100.2774	0.5320	**0.2940**	**0.6728**	0.6378	0.8346	**0.8731**
9. 1lcx	−96.6685	**−97.2231**	1.2500	**0.8120**	0.3523	**0.3529**	**0.7269**	0.6808
10. 3bu3	−101.1031	**−104.0929**	**1.5160**	1.6700	**0.3080**	0.3024	**0.6467**	0.6198
11. 1gjf	−104.9334	**−106.6305**	**1.0220**	1.3300	0.5593	**0.6295**	**0.7857**	0.7821
12. 1k43	−86.3976	**−88.8112**	1.7560	**1.4680**	0.2822	**0.2883**	**0.6143**	0.5821
13. 1a13	−40.1823	**−48.5602**	1.9200	**1.3460**	0.3665	**0.3975**	0.7286	**0.7357**
14. 1dep	−140.5410	**−142.4070**	1.1860	**1.0420**	0.6094	**0.6249**	0.9367	**0.9533**
15. 2bta	−180.0959	**−181.545**	1.4880	**1.1420**	0.2301	**0.2452**	**0.5867**	0.5733
16. 1nkf	−89.6251	**−90.9786**	1.1080	**0.9380**	0.3099	**0.3108**	**0.6750**	0.6563
17. 1le3	−76.2782	**−94.4471**	2.2440	**2.2140**	0.2467	**0.2513**	0.5674	**0.5780**
18. 1pgbF	−96.8106	**−102.0640**	1.5000	**1.3280**	0.2481	**0.2693**	0.5705	**0.6193**
19. 1niz	−86.4210	**−94.8362**	1.6260	**1.1780**	0.2466	**0.2469**	**0.5214**	0.4929
20. 1e0q	−50.4039	**−65.4440**	**1.4460**	1.9880	**0.2347**	0.2149	**0.5000**	0.4824
21. 1wbr	−148.1450	**−152.6280**	**1.6240**	1.7820	**0.3127**	0.3123	**0.6566**	0.6558
22. 1rpv	−306.9186	**−310.1344**	**0.7480**	0.8240	0.4976	**0.5332**	0.7412	**0.8617**
23. 1b03	**−119.8978**	−113.3539	1.6300	**1.2060**	0.2182	**0.2793**	0.4472	**0.4639**
24. 1pef	**−127.6188**	−123.9900	0.3580	**0.2920**	0.7168	**0.7260**	**0.9722**	0.9639
25. 1l2y	−133.7934	**−152.4246**	2.2340	**1.6100**	0.3089	**0.3486**	**0.6450**	0.6250
26. 1du1	−196.6084	**−198.5546**	1.4640	**1.2340**	0.2958	**0.3050**	**0.6400**	0.6025
27. 1pei	−197.7513	**−198.8485**	**1.0140**	1.1420	0.4075	**0.4135**	0.7335	**0.7443**
28. 1wz4	−157.5255	**−163.4536**	2.5540	**2.5100**	0.2720	**0.2849**	0.5065	**0.5500**
29. 1yyb	**−280.2210**	−266.9556	1.8080	**1.3340**	0.4495	**0.4569**	0.7327	**0.**7327
30. 1by0	−274.7173	**−274.8341**	**1.4480**	1.5940	0.4890	**0.5147**	**0.7426**	0.7352

The bold numbers in the table indicate the best value for each metric.

**Table A2:** Result values of GRSA2-FCNN and GRSABio-FCNN from 31 to 60 instances, using the metrics: Energy, RMSD, TM-score, and GDT-TS.

	Energy Ave.		RMSD Ave.		TM-Score Ave.		GDT-TS Ave.	
Instances	GRSA2-FCNN	GRSABio-FCNN	GRSA2-FCNN	GRSABio-FCNN	GRSA2-FCNN	GRSABio-FCNN	GRSA2-FCNN	GRSABio-FCNN
31. 1t0c	−124.9148	**−147.2932**	2.9060	**2.5640**	**0.2628**	0.2352	0.4290	**0.4403**
32. 2gdl	−225.0245	**−231.8165**	1.8120	**1.2400**	0.3113	**0.3287**	0.4000	**0.4306**
33. 2l0g	−78.1438	**−135.3045**	3.3700	**2.3900**	0.2650	**0.2828**	0.5831	**0.6221**
34. 2bn6	−266.2744	**−267.7155**	2.4160	**1.9140**	0.3556	**0.3617**	0.7468	**0.7595**
35. 2kya	−70.0008	**−151.1348**	2.9960	**2.3800**	0.2413	**0.2882**	0.3074	**0.3309**
36. 1wr3	−158.1745	**−230.0375**	**2.9820**	3.0180	0.2445	**0.2510**	0.3069	**0.3375**
37. 1wr4	−211.1636	**−214.6153**	**2.6900**	3.2220	0.2439	**0.2565**	0.2833	**0.3514**
38. 1e0m	−188.4646	**−200.8406**	**3.1520**	3.4580	**0.2442**	0.2430	**0.3135**	0.3014
39. 1yiu	−251.4423	**−252.6822**	3.2560	**3.0840**	0.2443	**0.2547**	0.3000	**0.3284**
40. 1e0l	−177.8004	**−223.3877**	2.8620	**2.7660**	0.2457	**0.2564**	0.2865	**0.2906**
41. 1bhi	−148.8985	**−175.5776**	2.8080	**2.7040**	**0.3024**	0.2421	0.3619	**0.3908**
42. 1jrj	−131.3705	**−210.8226**	2.5320	**1.9880**	**0.3114**	0.3972	0.3487	**0.3987**
43. 1i6c	−153.8447	**−185.2439**	**3.3920**	3.4680	0.2439	**0.2550**	0.2923	**0.3141**
44. 1bwx	−326.6206	**−330.6057**	2.3680	**2.3360**	0.4760	**0.4983**	0.5231	**0.5949**
45. 2ysh	−175.8431	**−183.8752**	3.3320	**3.1820**	0.2494	**0.2558**	0.2950	**0.3037**
46. 1wr7	−224.9640	**−233.5755**	3.0100	**2.8900**	**0.2635**	0.2514	0.3122	**0.3293**
47. 1k1v	−165.7520	**−284.3404**	2.6640	**2.5180**	0.3060	**0.3542**	**0.2097**	0.2073
48. 2hep	−121.0992	**−184.8579**	**2.7580**	2.9820	0.3117	**0.3608**	0.3083	**0.4024**
49. 2dmv	−189.2054	**−191.4439**	**3.0380**	3.5340	**0.2575**	0.2456	**0.2907**	0.2698
50. 1res	−144.2967	**−216.6926**	2.8700	**2.8560**	0.3026	**0.3156**	**0.3140**	0.3093
51. 2p81	−413.1129	**−430.3809**	2.6440	**2.5140**	**0.4001**	0.3840	0.3375	**0.3966**
52. 1ed7	−44.4465	**−179.0525**	**3.2240**	3.2780	0.2680	**0.2774**	0.2948	**0.3051**
53. 1f4i	−271.4136	**−384.3786**	**2.7100**	2.7640	0.3436	**0.3615**	0.3611	**0.3800**
54. 2l4j	−79.2959	**−196.8989**	**3.3260**	3.3940	0.2546	**0.2582**	0.2800	**0.2841**
55. 1qhk	−107.7690	**−199.9758**	**3.4340**	3.6260	0.2731	**0.2861**	0.2393	**0.2457**
56. 1dv0	−265.8274	**−338.6817**	**2.6600**	2.9340	**0.3177**	0.3040	0.3622	**0.3678**
57. 1pgy	−374.6846	**−390.3784**	2.4480	**2.3540**	**0.3353**	0.3283	**0.3851**	0.3553
58. 1e0g	−107.2451	**−238.9335**	3.8200	**3.3260**	0.2731	**0.3225**	0.2656	**0.2729**
59. 1ify	−315.1808	**−346.1995**	3.3100	**2.9820**	0.3319	**0.3698**	0.3983	**0.4438**
60. 1nd9	−171.3091	**−227.3267**	3.5260	**3.2820**	0.2654	**0.3022**	0.2541	**0.2979**

The bold numbers in the table indicate the best value for each metric.

**Table A3:** Result values of approaches from 1 to 30 instances, using the metrics: RMSD, TM-score, and GDT-TS.

	RMSD Ave.					TM-Score Ave.					GDT-TS Ave.				
Instances	GRSA2-FCNN	GRSABio-FCNN	GRSA2-FCNN	GRSA2-FCNN	GRSA2-FCNN	GRSA2-FCNN	GRSA2-FCNN	PEP-FOLD3	AlphaFold2	I-TASSER	GRSA2-FCNN	**GRSABio-FCNN**	**PEP-FOLD3**	**AlphaFold2**	**I-TASSER**
1. 1egs	0.7620	0.9120	**0.6600**	0.6760	-	0.3615	**0.3744**	0.2629	0.2976	-	0.6556	0.6666	**0.6722**	0.5722	-
2. 1uao	1.6200	**0.6020**	1.2820	1.0140	1.3200	0.3932	0.4241	0.4565	**0.4866**	0.3825	0.6900	0.6550	0.9000	**0.9300**	0.8750
3. 1l3q	1.0740	0.9500	2.0200	**0.1641**	0.2391	0.2629	**0.3013**	0.2391	0.1275	0.1979	0.6041	**0.6667**	0.6333	0.3416	0.4333
4. 2evq	1.9440	1.7360	0.8800	**0.3520**	1.6120	0.3182	0.3646	0.4503	**0.7171**	0.2443	0.7042	0.6958	0.9208	**1.0000**	0.6750
5. 1le1	1.7020	1.6680	1.1360	**0.6320**	0.9800	0.3179	0.3047	0.3510	**0.4673**	0.3539	0.6250	0.5834	0.8083	**0.9875**	0.9167
6. 1in3	1.2140	0.7480	**0.6120**	1.1920	1.0400	0.4393	**0.5034**	0.4071	0.4443	0.4201	0.5958	0.5875	0.6667	0.6375	**0.6875**
7. 1eg4	1.5160	1.3840	**0.7740**	1.7320	1.6240	0.3456	**0.3545**	0.2488	0.2913	0.2137	0.5962	0.5385	0.4731	**0.7346**	0.5961
8. 1rnu	0.5320	**0.2940**	0.8280	0.4040	0.8900	**0.6728**	0.6378	0.6355	0.5862	0.4110	0.8346	0.8731	0.8884	**0.9231**	0.7346
9. 1lcx	1.2500	**0.8120**	1.2580	1.5840	1.4900	0.3523	0.3529	0.3402	**0.3644**	0.3375	0.7269	0.6808	0.7308	**0.7654**	0.7308
10. 3bu3	1.5160	1.6700	**1.4100**	1.9600	1.6020	**0.3080**	0.3024	0.2553	0.1982	0.2266	**0.6467**	0.6198	0.5675	0.4406	0.5037
11. 1gjf	1.0220	1.3300	**0.9120**	0.9580	2.0100	0.5593	**0.6295**	0.6218	0.6014	0.6169	0.7857	0.7821	0.8214	0.8036	**0.8393**
12. 1k43	1.7560	1.4680	1.5660	1.3080	**0.9875**	0.2822	0.2883	0.3652	**0.3837**	0.3347	0.6143	0.5821	0.8321	**0.9215**	0.7188
13. 1a13	1.9200	1.3460	**1.2900**	1.4120	1.3900	0.3665	**0.3975**	0.3484	0.3019	0.3339	0.7286	0.7357	0.7322	**0.7536**	0.7500
14. 1dep	1.1860	1.0420	**0.8600**	0.9560	1.2800	0.6094	**0.6249**	0.5444	0.4521	0.5887	0.9367	**0.9533**	0.9389	0.8233	0.9000
15. 2bta	1.4880	**1.1420**	2.4260	1.2100	2.4600	0.2301	**0.2452**	0.1866	0.1986	0.1750	0.5867	0.5733	0.5933	**0.6100**	0.5833
16. 1nkf	1.1080	0.9380	**0.6500**	1.3860	1.2680	0.3099	**0.3108**	0.2612	0.2474	0.2899	0.6750	0.6563	0.5969	0.5688	**0.7781**
17. 1le3	2.2440	2.2140	2.1460	**0.9120**	1.2300	0.2467	0.2513	0.2060	**0.4101**	0.3090	0.5674	0.5780	0.4246	**0.7412**	0.6313
18. 1pgbF	1.5000	1.3280	1.6900	**1.1520**	1.4420	0.2481	0.2693	0.2504	**0.3418**	0.3386	0.5705	0.6193	0.5161	0.6621	**0.6918**
19. 1niz	1.6260	**1.1780**	1.3000	1.9060	1.9420	0.2466	0.2469	**0.2867**	0.1749	0.2451	**0.5214**	0.4929	0.3688	0.4464	0.4429
20. 1e0q	1.4460	1.9880	1.3300	1.5700	**0.9400**	0.2347	0.2149	0.3080	0.2530	**0.3223**	0.5000	0.4824	0.8382	0.7765	**0.8971**
21. 1wbr	1.6240	1.7820	1.0940	1.6520	**0.8200**	**0.3127**	0.3123	0.2670	0.2927	0.2835	**0.6566**	0.6558	0.5503	0.5670	0.5792
22. 1rpv	**0.7480**	0.8240	0.9520	0.6080	1.2600	0.4976	**0.5332**	0.3826	0.4779	0.5778	0.7412	0.8617	0.8264	0.8588	**0.8676**
23. 1b03	1.6300	**1.2060**	1.4900	1.9720	2.2520	0.2182	**0.2793**	0.2502	0.2733	0.1436	0.4472	0.4639	0.4389	**0.4889**	0.4000
24. 1pef	0.3580	**0.2920**	0.5780	0.3880	0.3400	0.7168	**0.7260**	0.6805	0.7225	0.6493	0.9722	0.9639	0.9694	**0.9778**	0.9722
25. 1l2y	2.2340	1.6100	2.0280	**0.7540**	2.1100	0.3089	0.3486	0.3322	**0.4751**	0.1471	0.6450	0.6250	0.7300	**0.9650**	0.5175
26. 1du1	1.4640	**1.2340**	1.4400	1.6800	1.6750	0.2958	**0.3050**	0.2505	0.2789	0.2704	0.6400	0.6025	0.6575	**0.6650**	0.6500
27. 1pei	**1.0140**	1.1420	1.6120	1.1640	1.7620	0.4075	0.4135	0.3472	**0.4180**	0.3682	0.7335	0.7443	0.7156	**0.8097**	0.7522
28. 1wz4	2.5540	2.5100	**1.9960**	2.8060	2.2820	0.2720	**0.2849**	0.2429	0.2596	0.2508	0.5065	0.5500	0.5217	**0.5957**	0.5587
29. 1yyb	1.8080	**1.3340**	1.7080	1.6460	2.1680	0.4495	**0.4569**	0.3849	0.4340	0.3779	0.7327	0.7327	0.7148	**0.7558**	0.6444
30. 1by0	**1.4480**	1.5940	1.5750	1.7700	1.8200	0.4890	**0.5147**	0.4682	0.4903	0.4540	0.7426	0.7352	0.7732	0.7871	**0.8056**

The bold numbers in the table indicate the best value for each metric.

**Table A4:** Result values of approaches from 31 to 60 instances, using the metrics: RMSD, TM-score, and GDT-TS.

	RMSD Ave.							TM-Score Ave.							GDT-TS Ave.						
Instances	GRSA2	GRSAB	PEP	Alpha	I-TAS	Ros	Top	GRSA2	GRSB	PEP	Alpha	I-TAS	Ros	Top	GRSA2-FCNN	GRSABio-FCNN	PEP-FOLD3	AlphaFold2	I-TASSER	Rosetta	TopModel
31. 1t0c	2.9060	**2.5640**	2.8160	2.8860	3.1440	2.7017	3.0733	0.2628	0.2352	0.2070	0.1981	0.2260	**0.3085**	0.2002	0.4290	0.4403	0.3806	0.3000	0.4726	**0.4745**	0.3199
32. 2gdl	1.8120	**1.2400**	1.8180	2.5920	2.3800	2.2600	1.8120	0.3113	0.3287	0.3246	0.3147	0.3815	0.3151	**0.3970**	0.4000	0.4306	0.4290	0.5323	**0.6145**	0.3887	0.4790
33. 2l0g	3.3700	2.3900	2.2820	1.5320	1.6880	2.1860	**1.4720**	0.2650	0.2828	0.5248	0.6485	0.5725	0.5895	**0.6568**	0.5831	0.6221	0.5856	0.7237	0.6389	0.6579	**0.7330**
34. 2bn6	2.4160	1.9140	2.0800	1.9820	1.9525	1.8900	**1.5760**	0.3556	0.3617	0.3214	0.5445	0.3976	**0.5654**	0.5337	0.7468	**0.7595**	0.3928	0.5968	0.4529	0.6248	0.5957
35. 2kya	2.9960	2.3800	1.8120	2.6440	2.8200	**1.7460**	1.9260	0.2413	0.2882	0.3127	0.2858	0.3193	0.3586	**0.5300**	0.3074	0.3309	0.3514	0.4132	0.4250	0.4588	**0.6853**
36. 1wr3	2.9820	3.0180	2.2280	1.8440	1.7600	1.9120	**1.0240**	0.2445	0.2510	0.4618	0.7041	0.6583	0.6228	**0.7713**	0.3069	0.3375	0.6472	0.8806	0.8472	0.8236	**0.9194**
37. 1wr4	2.6900	3.2220	2.2040	1.6440	1.5867	1.4700	**1.3840**	0.2439	0.2565	0.5142	0.7339	**0.7376**	0.7226	0.7009	0.2833	0.3514	0.6861	**0.8861**	0.8819	**0.8861**	0.8667
38. 1e0m	3.1520	3.4580	2.0820	**1.5640**	1.8900	1.9860	1.6040	0.2442	0.2430	0.4821	0.6956	0.6973	0.6548	**0.7173**	0.3135	0.3014	0.6446	0.8595	0.8497	0.8257	**0.8702**
39. 1yiu	3.2560	3.0840	2.3620	1.5600	1.5367	**1.1460**	1.3780	0.2443	0.2547	0.4649	0.7308	0.7188	**0.7417**	0.6707	0.3000	0.3284	0.6608	**0.9000**	0.8919	0.8865	0.8486
40. 1e0l	2.8620	2.7660	2.2140	1.7660	1.7300	**1.1700**	1.8060	0.2457	0.2564	0.4943	**0.7152**	0.6733	0.6734	0.6246	0.2865	0.2906	0.6703	**0.8568**	0.8216	0.8054	0.7824
41. 1bhi	2.8080	2.7040	2.7460	2.2140	2.2680	2.1080	**2.1200**	0.3024	0.2421	0.3397	**0.6673**	0.6313	0.6356	0.5986	0.3619	0.3908	0.4553	**0.8171**	0.7803	0.7908	0.7539
42. 1jrj	2.5320	1.9880	2.0360	**1.8240**	2.2040	2.1200	1.8260	0.3114	0.3972	0.4929	**0.6687**	0.5390	0.5303	0.6032	0.3487	0.3987	0.5923	**0.8192**	0.6756	0.6833	0.7475
43. 1i6c	3.3920	3.4680	2.4920	2.2940	2.5040	2.5460	**2.2760**	0.2439	0.2550	0.4219	0.5581	0.4454	**0.5625**	0.5304	0.2923	0.3141	0.5680	**0.7077**	0.5769	0.7025	0.6910
44. 1bwx	2.3680	2.3360	2.2060	2.0940	**1.7100**	1.8140	2.4980	0.4760	0.4983	0.4727	0.4979	**0.5854**	0.5614	0.4965	0.5231	0.5949	0.5539	0.5679	**0.7051**	0.6833	0.5859
45. 2ysh	3.3320	3.1820	**2.0500**	2.3100	2.0700	2.1960	2.2700	0.2494	0.2558	0.4712	**0.5880**	0.5651	0.5854	0.5550	0.2950	0.3037	0.6100	0.7287	**0.7375**	0.7200	0.6988
46. 1wr7	3.0100	2.8900	2.0700	**1.4080**	1.2980	1.6080	1.3920	0.2635	0.2514	0.5284	0.7329	**0.7387**	0.6949	0.6776	0.3122	0.3293	0.6500	0.8439	**0.8464**	0.8195	0.7890
47. 1k1v	2.6640	2.5180	2.2120	0.9500	1.3500	1.2600	**1.2220**	0.3060	0.3542	0.5101	**0.8277**	0.7702	0.7452	0.7558	0.2097	0.2073	0.2805	0.2756	0.2805	0.2781	**0.2854**
48. 2hep	2.7580	2.9820	2.3020	2.0280	1.9550	**1.9360**	2.2400	0.3117	0.3608	0.5372	0.6374	**0.6421**	0.6189	0.5978	0.3083	0.4024	0.7107	**0.7940**	0.7828	0.7679	0.7619
49. 2dmv	3.0380	3.5340	1.9600	**1.7580**	2.1060	1.9720	1.9260	0.2575	0.2456	0.4756	0.6194	**0.6651**	0.6509	0.5877	0.2907	0.2698	0.6105	0.7430	**0.7919**	0.7651	0.7256
50. 1res	2.8700	2.8560	2.1720	1.9760	1.7700	**1.7360**	1.9520	0.3026	0.3156	0.4512	0.6295	0.6040	0.6192	**0.6330**	0.3140	0.3093	0.5756	**0.7663**	0.7442	0.7372	0.7616
51. 2p81	2.6440	2.5140	2.4400	2.2880	2.2420	2.0400	**1.9120**	0.4001	0.3840	0.4694	**0.5795**	0.5396	0.4943	0.4925	0.3375	**0.3966**	0.3137	0.3398	0.3395	0.3398	0.3398
52. 1ed7	3.2240	3.2780	3.4340	1.3380	1.4267	1.5800	**1.3980**	0.2680	0.2774	0.3306	**0.7957**	0.7954	0.7113	0.7680	0.2948	0.3051	0.3689	**0.8879**	0.8877	0.7938	0.7338
53. 1f4i	2.7100	2.7640	2.4500	1.5500	1.4533	**1.2820**	1.4100	0.3436	0.3615	0.4075	0.8023	0.7849	**0.8332**	0.8119	0.3611	0.3800	0.4489	0.8956	0.8871	**0.9156**	0.9044
54. 2l4j	3.3260	3.3940	2.5620	2.2180	2.4340	2.4040	**2.0200**	0.2546	0.2582	0.3802	**0.5459**	0.5129	0.4988	0.5395	0.2800	0.2841	0.3734	**0.6005**	0.5642	0.5487	0.5935
55. 1qhk	3.4340	3.6260	3.2800	2.3740	2.6660	2.4340	**2.2700**	0.2731	0.2861	0.2881	**0.6122**	0.4555	0.5793	0.5707	0.2393	0.2457	**0.2957**	0.2351	0.2394	0.2319	0.2340
56. 1dv0	2.6600	2.9340	1.9400	**1.2860**	1.4600	1.6640	1.5020	0.3177	0.3040	0.3980	0.7689	**0.7853**	0.7551	0.7608	0.3622	0.3678	0.3734	0.5011	0.4752	0.4734	**0.5078**
57. 1pgy	2.4480	2.3540	2.4000	2.3800	**0.4200**	2.4000	2.5060	0.3353	0.3283	0.5053	0.6338	**0.9662**	0.6386	0.5679	0.3851	0.3553	0.6138	0.7575	**0.9947**	0.7415	0.6979
58. 1e0g	3.8200	3.3260	2.9520	1.8240	1.8300	**1.8200**	3.3580	0.2731	0.3225	0.4487	0.7396	**0.7500**	0.6981	0.2985	0.2656	0.2729	0.5656	0.8448	**0.8594**	0.8104	0.2915
59. 1ify	3.3100	2.9820	2.7800	1.5020	2.1600	**1.1520**	1.7460	0.3319	0.3698	0.3635	0.8029	0.7123	**0.8485**	0.7545	0.3983	0.4438	0.4362	0.8799	0.8113	**0.9376**	0.7209
60. 1nd9	3.5260	3.2820	3.1580	**2.7000**	2.8550	2.9820	3.2020	0.2654	0.3022	0.4018	**0.4789**	0.4628	0.2540	0.4169	0.2541	0.2979	0.4245	**0.4827**	0.4286	0.4368	0.4225

The bold numbers in the table indicate the best value for each metric. The abbreviations are as follows GRSA2 refers to GRSA2-FCNN, GRAB to GRSABio-FCNN, PEP to PEP-FOLD3, I-TAS to I-TASSER, Ros to Rosetta, and Top to TopModel.
